# Existing racial and ethnic representation on top management teams predicts subsequent top management team appointments that preserve the demographic status quo

**DOI:** 10.1371/journal.pone.0357710

**Published:** 2026-09-08

**Authors:** Christopher W. Bauman, John Morton, Ben Lourie, Philip Bromiley

**Affiliations:** 1 Paul Merage School of Business, University of California Irvine, Irvine, California, United States of America; 2 College of Business Administration, California State Polytechnic University, Pomona, California, United States of America; Universiti Brunei Darussalam, BRUNEI DARUSSALAM

## Abstract

Top management teams (TMTs) are considerably less diverse than employees at other levels and the general population. Using over 48,000 firm–year observations of executives across 3,807 firms from 1992 to 2017, we find robust evidence that the existing racioethnic composition of TMTs predicts subsequent TMT appointments that preserve the racioethnic status quo. Specifically, having a racioethnic minority group member on a TMT has a negative association with appointing another member of the same group, and the departure of a racioethnic minority group member from a TMT has a strong positive association with an appointment of a member of that group. However, having a CEO from a racioethnic minority group has a positive association with an appointment from the CEO’s racioethnic group. Moreover, the associations with departures and CEO racioethnicity appear stronger in more recent years (2005–2017) than in the past (1992–2004). Theories of diversity in the upper echelon of firms that focus on the role of stereotypes cannot fully explain these effects. The results are consistent with a status uncertainty account we derive from theories of intergroup dynamics. The results also show that TMT appointment decisions hinge on finer-grained distinctions among racioethnic categories than typically used by researchers and the US Census.

## Introduction

Diversity can exist along many dimensions (e.g., tenure, functional background, gender), but racioethnic diversity is a particular emphasis in the media [1], practitioner publications [2], and research on organizational behavior [3]. However, studies of top management teams (TMTs) to date omit racioethnic diversity [4]. This absence of research on the racioethnic diversity of TMTs may be due to a lack of racioethnic diversity in these positions, which has only recently and gradually begun to change. A recent survey of 222 companies in the United States (representing over 12 million employees) found that only 13% of C-suite executives were people of color [5]. While many firms express interest in increasing racioethnic diversity across all levels of the organization, TMTs remain much less racioethnically diverse than employees at other levels and the general population. Therefore, understanding factors associated with TMT racioethnic composition is important to both scholarship and practice.

We examine how the existing racioethnic composition of TMTs predicts subsequent appointments of racioethnic minority group members to TMTs to shed light on why TMTs remain relatively homogeneous in terms of racioethnicity despite widespread institutional pressure to increase diversity [2,6,7]. Our approach is novel because we focus on how the racioethnic composition of TMTs unfolds longitudinally as opposed to studying the composition cross-sectionally, and we articulate how intergroup dynamics can account for the association between the existing racioethnic composition of the TMT and subsequent appointments. Our perspective complements and extends theories of the antecedents of TMT and board diversity that largely focus on the role of stereotypes and cannot fully explain the patterns we observe.

Drawing from social identity theory and prior research on tokenism, we theorize that the prospect of adding underrepresented minority group members to the TMT raises status concerns that influence appointment decisions. Demographic dissimilarity can trigger individuals’ concerns about their status relative to other individuals on the team and the status of their team relative to other teams [8–12]. Majority group members (e.g., whites in US firms) in high status positions may worry that adding minority group members to the team would decrease their status [13–15]. Moreover, members of minority groups (e.g., racioethnic minority group members) risk losing status for advocating for people like themselves [16–18], or they may seek to preserve their unique position as the only member of their minority group on the team and the benefits associated with it [19–22]. Thus, when considering whom to appoint to the TMT, status concerns may induce a tendency to appoint individuals who would maintain the demographic status quo.

Using data that includes over 48,000 firm–year observations of executives across 3,807 firms covered by ExecuComp from 1992 to 2017, we find that the existing racioethnic composition of the TMT predicts the racioethnicity of executives subsequently appointed to the TMT. Specifically, having a racioethnic minority group member on a TMT has a negative association with appointing another member of the same group. Also, the departure of a racioethnic minority group member from a TMT has a strong positive association with an appointment of a member of that group. Together, these results appear to represent status quo bias because they suggest that firm leaders—including the Board of Directors who have formal power to make appointments and other parties who influence the decisions—resist both increasing and decreasing representation of racioethnic minority groups on the TMT. Researchers frequently use such evidence of resistance to change to document status quo bias in the decision-making literature (i.e., a reversal test; see [23]).

One important caveat to the pattern of results described above exists when the CEO is a racioethnic minority group member. Although racioethnic minority groups are underrepresented among CEOs in our sample, we observe that having a CEO who is a racioethnic minority group member has a positive association with an appointment of members from the CEO’s racioethnic group to the TMT. This result is perhaps not surprising because people tend to affiliate with demographically similar others [24,25] and CEOs have considerable power in TMT appointment decisions [26]. However, it is striking that having a racioethnic minority group member on the TMT in any other role has the opposite effect. Taken together, the combined results strongly suggest that existing racioethnic representation on the TMT affects subsequent appointments. Also, the pattern of results is consistent with our status concerns account and are difficult to explain using alternative mechanisms discussed in the literature.

We aim to contribute to the literatures on TMT appointments and diversity in four main ways. First, we extend theories of the antecedents of TMT appointments by identifying a novel and important influence on them. We argue that the prospect of appointing people who would change the racioethnic composition of a TMT can create status uncertainty for those in leadership positions. Prior research on TMT appointment decisions focuses on institutional pressures to increase diversity and the tendency for majority group members to stereotype and derogate outgroup members. Our status uncertainty account involves individuals’ concerns about group-level dynamics within TMTs that operate alongside institutional pressures and stereotyping.

Second, we are the first to empirically examine how racioethnic representation on TMTs predicts subsequent TMT appointments. Understanding the role of racioethnicity in TMT appointment decisions has intrinsic value, and our results lend broader insight into the social dynamics of appointment decisions when multiple salient categories (i.e., subgroups) exist within a demographic dimension. Although research on gender has yielded important insights about how demographic categories can influence TMT appointments, researchers typically treat gender as a binary variable. Therefore, research on gender can only illuminate the dynamics between a dominant group and a single subordinate group, whereas research on racioethnicity can explore intergroup relations when multiple salient categories exist within a given dimension. Moreover, racioethnicity and gender are distinct demographic dimensions that differ in important ways and warrant separate consideration [27]. Our results also indicate that the associations between racioethnicity and TMT appointments appear to be strengthening rather than declining in recent years.

Third, because racioethnicity involves many categories, it allows us to explore questions about the granularity of the categories people use when making appointment decisions. We examine differences across traditional racioethnic categories (e.g., Asian, African American, Hispanic), but we also explore the utility of considering finer-grained categories that reflect more specific groups (e.g., East Asian vs. Indian). Although many discussions of racioethnicity use superordinate racioethnic categories (e.g., Asian), we find that finer-grained category membership predicts TMT appointments. Thus, our research also makes a novel contribution because it indicates that people categorize others at a more granular level than is typically used to describe racioethnic groups.

Fourth, we provide empirical evidence that CEO racioethnicity is associated with the selection of new TMT members. Prior research on gender suggests that when the CEO of a firm is a woman, the proportion of women decreases on TMTs [20] but increases on boards [28]. We find that CEO racioethnicity increases the likelihood of adding another individual of the same racioethnicity to the TMT, even though we find that the representation of a given racioethnicity in a TMT generally decreases the likelihood of subsequent additions from the same ethnic group. This result aligns with the view that unlike most TMT members, CEOs tend to have the political capital necessary to advocate for individuals like themselves [26,29,30]. Thus, our research suggests that CEOs are in a unique position to increase TMT racioethnic diversity.

### Racioethnic minority groups

Societal pressure to include diversity in the workplace may reference a variety of demographic dimensions, but the primary focus is on gender and racioethnicity [1,2]. Existing theory and empirical research on corporate governance and appointment decisions examines several important demographic dimensions (e.g., age, education, functional experience, gender) but not racioethnicity [4]. Racioethnicity is an especially influential social category [31–33]. People spontaneously use racioethnicity to code and understand social situations [31], and racioethnicity is particularly salient and important to members of minority groups, even in situations that do not call attention to issues of racioethnicity in the minds of majority group members [34,35]. Moreover, similarity in some demographic categories often compensates for differences across other categories, but perceived racioethnic dissimilarity is more difficult to overcome [33]. In short, the effect of racioethnicity on TMT appointment decisions is an important, distinct, and understudied phenomenon.

We operationalize racioethnic minority groups in the context of TMT membership in US firms as non-European ancestries (African, African American, East Asian, Hispanic, Indian, Middle Eastern). We avoid the term “white” because it is a contested category [36]. For example, the US Census Bureau [37] defines white as “A person having origins in any of the original peoples of Europe, the Middle East, or North Africa.” However, many people with Middle East and North Africa ancestries do not identify as white, and they face more discrimination and prejudice than people with European ancestries [38,39].

Before proceeding, we briefly articulate the importance of examining the antecedents of TMT appointment decisions, given the availability of high-quality studies of gender and potential similarities across these demographic dimensions. For example, at least one study considers the appointment of women to TMTs [20], and a considerable amount of work has considered the appointments of women to corporate boards [28,40–42]. However, findings about boards may not generalize to TMTs due to their different functions, legal status, and appointment mechanisms, and findings about gender may not generalize to racioethnicity.

The legal underpinning of boards of directors differs dramatically from that of TMTs [43]. To begin with, a substantial set of legal and procedural rules govern boards of directors, but a TMT is a concept with no legal foundation. In practice, academics often identify the TMT as the most highly paid executives (those whose compensation must be reported) but being most highly paid does not indicate the set of individuals operates as a team or makes any decisions collectively. Corporate documents requiring shareholder approval determine the number of directors, but the number of TMT members can be changed without such approval (subject to reporting compensation on a minimum number of executives). Board members must be elected by the shareholders, but CEOs (and sometimes boards) hire and fire TMT members at will. In law, TMT members are employees of the firm, but board members are not. In addition, boards and board members have no direct responsibility for running any part of the corporation whereas TMT members have such responsibilities. Thus, understanding how boards operate may not generalize to TMTs.

Although racioethnicity and gender may operate similarly in some ways, they differ in important ways. First, although women and members of racioethnic minority groups face stigma and discrimination, each social group has a unique history and set of contemporary challenges that may influence the way firm leaders and the general public conceptualize and address issues relative to a particular group [44,45]. That is, although women and racioethnic minority group members face discrimination, they are treated very differently in many contexts. For example, the US legal system treats white women differently than it treats certain racioethnic minority groups (and it treats different racioethnic minority groups differently). Second, as mentioned above, racioethnicity is structurally more complex because it includes more categories than gender, which affects the social dynamics among groups in several ways. For example, based simply on the mathematics of combinations, the sheer number of intergroup considerations increases dramatically as the number of categories increases. Also, as the number of groups increases, so too does the potential for coalitions to form [46]. In short, it is important to examine TMT racioethnicity for its own sake, and its complexity can shed light on phenomena that research on gender cannot capture.

### Antecedents to TMT appointments

Firm leaders face pressure to appoint more racioethnic minority group members to the TMT for both ethical and instrumental reasons. Many people believe it is unethical to discriminate on the basis of racioethnicity [7], yet people of color are underrepresented in firm leadership positions [5]. Also, considerable evidence indicates that demographic diversity can increase firm performance in some contexts [6,47,48]. However, at least two major factors may inhibit TMT appointments of racioethnic minority group members. The following sections explore these mechanisms.

### Stereotyping

Firm leaders may avoid appointing racioethnic minority group members to TMTs due to stereotyping and prejudice. Several theoretical perspectives maintain that people often perceive individuals from other demographic categories as outsiders and thus resist their inclusion in groups [21,25,49,50], but self-categorization theory is especially influential in the corporate governance literature [48,51,52]. Self-categorization theory asserts that people commonly categorize themselves and others into groups based on demographic characteristics, including racioethnicity [50,53]. Through categorization, people form group prototypes based on their conscious or unconscious beliefs about what features define the group. For example, many people may see being white and male as prototypical characteristics of TMT members because white men hold a substantial majority of TMT positions in the US. These social categorizations may cause those responsible for TMT appointment decisions to view minority group members as outsiders who would not fit the TMT.

People also tend to affiliate with and exhibit favoritism toward others who are demographically similar [24,54], which increases the likelihood that dominant group members see other dominant group members as the best candidates for leadership positions [55]. Moreover, stereotyping and social categorization can change the way people interpret behaviors. Behaviors that often advantage white men (e.g., advice seeking, ingratiation to CEOs) can backfire for women and racioethnic minority group members [56,57]. In short, surface-level characteristics may have a powerful influence on TMT appointments because they spontaneously activate stereotypes and trigger social categorizations that advantage majority group members and disadvantage minority group members. Thus, stereotyping and social categorization can inhibit firm leaders from appointing racioethnic minority group members to TMTs.

### Status Uncertainty

Although some have questioned the extent to which TMTs have the same features and operate like other teams (e.g., common purpose, mutual accountability, concrete goals), a large body of research on social identity theory shows that merely identifying group boundaries (i.e., who is and who is not a member of a group) is sufficient to influence people’s cognitions and behavior. Therefore, the common tendency for firm leaders and the general public to label the TMT as a team, even in a minimal sense, should be sufficient for the processes articulated by our theory to operate.

Drawing from the original formulation of social identity theory [49], we propose that status uncertainty is a second mechanism that has ties to stereotyping and social categorization but a distinct effect on TMT appointments. Although self-categorization theory (discussed above) is an integral part of what has become known as the broader social identity perspective, it focuses on intrapsychic processes, whereas the original formulation of social identity theory focuses on intergroup dynamics [49,54]. Importantly, the intrapsychic components of the social identity perspective (e.g., negative stereotypes, social categorizations) should produce a constant influence on each appointment decision, irrespective of the existing composition of the TMT. In contrast, the intergroup component of the social identity perspective should influence appointment decisions depending on whether or how an appointment would change the status quo. Thus, the intergroup perspective we articulate complements and extends prior work on TMT and board appointments that focuses on stereotyping.

Social identity theory states that a dominant coalition in an existing group may consciously or unconsciously feel threatened by the prospect of adding new members who could destabilize the status quo. Majority group members experience identity threat when events create uncertainty, challenge the status hierarchy in the group, or jeopardize the status of the group relative to other groups [8,24]. When experiencing identity threat, majority group members act to maintain the status quo, thereby protecting their identity, their position in the group, and the group’s position vis-à-vis other groups [58,59]. The potential addition of new members to the group can trigger identity threat, but negative stereotypes can exacerbate the identity threat; the prospect of adding new people to the group creates more uncertainty and more disruption to within- and across-group status hierarchies when negative stereotypes exist about the prospective members’ demographics groups. Thus, negative stereotypes operating at the intrapsychic level aggravate social dynamics and identity threat that operate at the intergroup level.

Additionally, the prospect of having multiple members of a racioethnic minority group on a TMT may be especially likely to trigger identity threat and prompt firm leaders to resist appointments that change the status quo. Given that coalitions are more likely to form among individuals from the same rather than different racioethnic groups [60,61], firm leaders may be especially reluctant to appoint a second member of a racioethnic minority group. Moreover, individuals experience much lower conformity pressure when they join a group with some members (rather than none) who share their demographic characteristics [62]. As a result, firm leaders may view racioethnicity as the potential basis for a faultline that could change the dynamics of the group [63]. Taken together, firm leaders may accept having one representative from a given racioethnic minority group but the potential for the development of coalitions and faultlines may make them resist adding more than one.

In summary, TMTs remain much less racioethnically diverse than employees at other levels and the general population despite societal pressures to increase diversity. To gain insight into this phenomenon, we examine how the existing racioethnic composition of TMTs influences subsequent appointments of racioethnic minority group members to TMTs. We propose that two mechanisms—stereotyping and status uncertainty—influence TMT appointment decisions in ways that reinforce the racioethnic status quo. Specifically, stereotyping will generally inhibit appointments of racioethnic minority group members. Status uncertainty also will inhibit appointments of racioethnic minority group members in general but especially appointments of racioethnic minority group members whose group is already represented on the TMT.

**Hypothesis 1.** *The presence of an individual from a racioethnic minority group on a TMT will be negatively associated with the probability of adding another individual from that racioethnic group to the TMT.*

In addition to examining how the existing racioethnic composition of the TMT relates to decisions about new appointments, we also consider how the racioethnicity of those who depart TMTs influences decisions about who will replace them. Observing that firms tend to replace a departing TMT member with an individual from the same racioethnic group as the one leaving would provide further evidence of the role of intergroup dynamics because it would suggest a resistance to decreases in representation of a given racioethnic group. In conjunction with evidence of a negative association between representation of a given racioethnic group and appointments of members of the same racioethnic group (i.e., Hypothesis 1), finding a positive association between the departure of a member of a given racioethnic group and appointments of members of the same racioethnic group would constitute a reversal test. Reversal tests show aversion to changes in either direction on a given parameter and are frequently used in the decision-making literature to demonstrate status quo bias (see [23]).

The social identity perspective (the basis for both the stereotyping and status uncertainty arguments above) maintains that people often use demographic differences as indicators of social group membership and tend to perceive members of outgroups in terms of their demographic categories rather than as individuals [49,50,54, see also 21]. Therefore, firm leaders may expect that replacing a departing TMT member with another individual from the same racioethnic group would be less disruptive to the status quo than appointing either a majority group member or someone from a different racioethnic minority group.

Prior research on gender finds similar replacement effects when women leave corporate boards [64]. However, because research treats gender as a binary variable, it cannot test whether these effects reflect a narrow tendency to replace departing minority group members with members of the same group or a broader tendency to replace departing minority group members with members of any minority group. When dealing with a variable with many categories, firms have several ways to increase diversity (i.e., increase variety/heterogeneity; [65]); a firm dominated by any given racioethnicity can increase diversity by adding individuals from any racioethnic group not already represented (e.g., adding an African American, Hispanic, or East Asian). In this way, racioethnic and gender diversity differ because a male-dominated firm can only increase gender diversity by adding women.

In sum, if firm leaders are not attending to intergroup dynamics, then the racioethnic group membership of departing TMT members should not influence the likelihood of their replacements being from the same racioethnic group. If firm leaders are invested in the racioethnic status quo, then they should replace departing TMT members with someone from the same racioethnic minority group.

**Hypothesis 2.** *The departure of an individual from a racioethnic minority group from a TMT will be positively associated with the probability of appointing another individual from that racioethnic group to the TMT.*

### CEO racioethnicity and TMT appointments

The sections above examine how the racioethnic composition of the TMT as a whole may influence TMT appointments, but having a CEO who is a racioethnic minority group member may have a distinct effect on TMT appointments for three reasons. First, CEOs often have a strong influence on TMT appointments [26,29,30]. Racioethnic minority group members may worry that supporting someone of their racioethnic group could reflect badly on themselves and negatively affect their career trajectory [16]. However, CEOs who are racioethnic minority group members may worry less about such problems because they already hold the top position in the firm and their networks are likely to include people from their own racioethnic group [66]. Second, CEOs who are racioethnic minority group members may be less invested in the racioethnic status quo because they are less likely to experience identity threat from the prospect of adding a member of their own group [44] and their group is likely to be underrepresented in leadership positions [5]. Third, having a CEO who is a racioethnic minority group member signals that the firm values that racioethnic group and that members of that group can succeed in the firm [67]. Therefore, candidates from racioethnic minority groups may be more attracted to a company with a CEO from their own racioethnic minority group, which could enrich the pool of high-quality prospects from that racioethnic minority group. In sum, CEOs who are racioethnic minority group members can advocate for members of their own racioethnic minority group to join the TMT, they should be less invested in the status quo when it comes to limiting opportunities for members of their own racioethnicity group, and candidates from the same racioethnic minority group as the CEO may be more attracted to the role. Together, these factors may produce a positive effect of CEO racioethnicity on TMT appointments of people who are members of the same racioethnic group as the CEO.

**Hypothesis 3.** *Having a CEO who is a racioethnic minority group member positively influences the likelihood of TMT appointments of members of the CEO’s racioethnic group.*

## Method

The Institutional Review Board at the University of California, Irvine does not require and will not provide approval for this research because it involves secondary uses of identifiable private information that is publicly available and external of the University (Exempt Category 4). For the same reason, informed consent was not required.

### Data construction

Our sample includes all the top managers identified in ExecuComp between 1992 and 2017. ExecuComp extracts its data directly from the annual U.S. Securities and Exchange Commission Form DEF 14A (commonly accepted as the definitive proxy statement). Under U.S. Securities and Exchange Commission rules, publicly traded companies must disclose detailed compensation packages for their “Named Executive Officers” each fiscal year, which includes the Chief Executive Officer, Chief Financial Officer, and minimally the next three most highly compensated executive officers. Prior research using the ExecuComp database operationalized TMTs as the most highly paid managers [20,68–72], and over 80% of the manager–years referred to individuals with titles that researchers have previously used to identify TMTs using other sources (e.g., CEO, COO, President, CFO). Collapsing the 305,000 person-firm-years to firm–years resulted in roughly 50,000 firm–year observations, although our estimates could only use years when someone on the TMT changed and values were available for all the included variables, resulting in over 20,000 firm-year observations.

To identify racioethnicity, we initially used a surname–ethnicity classifier developed by Ambekar, Ward, Mohammed, Male, and Skiena [73], which has been used extensively in previous research [74–76]. This classifier associates each manager’s name with a national origin, which resulted in an extremely large number of categories, many with few entries. We therefore aggregated the results into categories of racial minority groups that represented at least one percent of the sample (viz. African, East Asian, Hispanic, Indian, Middle Eastern). We then changed to the NamSor API v2 diaspora classifier, a commercial classifier with a proprietary machine learning algorithm that has predictive capabilities in the range 85%–95% (see www.namsor.com). NamSor also has been used in prior research [77–79]. We adopted NamSor for our primary analysis (see Table 1), and we discuss the results using the Ambekar et al [73] classifier in our section on additional analyses. Data from the two classifiers give similar results.

**Table 1:** Descriptive Statistics for Racioethnic Groups.

Variable Type	Racioethnic Groups	Number (person–firm–years)	% of Total (person–firm–years)
Foci of Hypotheses	African	4,565	1.50
	East Asian	6,470	2.12
	Hispanic	10,407	3.41
	Indian	4,219	1.38
	Middle Eastern	3,051	1.00
	African American	7,436	2.44
Control Categories	British	138,769	45.51
	Eastern European	9,091	2.98
	French	11,957	3.92
	German	41,093	13.48
	Italian	17,117	5.61
	Jewish	22,675	7.44
	Nordic	24,082	7.90
	Other	3,973	1.30
	Total	304,905	100.00

Others from groups with very low representation in the sample were omitted from analysis, including individuals classified as Greek, Macedonian, Kazakh, Moldova, Montenegrin, Native Hawaiian, and Trinidad/Tobago.

Name-based classifiers cannot accurately identify African Americans. We manually identified African American in the sample using (i) African American identifications in the ISS data on directors, (ii) Black Enterprise’s list of the top 300 Black executives in corporate America [80], and (iii) Black Enterprise’s list of Black board members for 2017 and 2018. Matching the data sets took substantial effort because the sources often refer to the same person with different variants of the name (e.g., John Smith and John William Smith could refer to the same person). When unsure about whether an individual matched, we checked online to see that the company listed in the secondary sources fit the individual in the full database. For some executives, we also checked online biographical information. For individuals we identified as African American, we replaced the racioethnicity indicated by the name classifier. Although we probably did not identify all African American executives in the data set, we are confident that all those identified as African American are in fact African American.

Manually identifying African Americans also provided an independent means to assess the validity of the results based on categories determined by the classifier algorithms. Theoretically, the results for all racioethnic minority groups should be in the same direction, even if the magnitude of the effects differ somewhat. In other words, for all racioethnic minority groups, we predict that representation on the TMT will have a negative effect on subsequent TMT appointments, and the departure of a racioethnic minority group member from the TMT will have a positive effect on subsequent TMT appointments of individuals from the same group. Therefore, if the classifier algorithms are performing adequately, the hand-coded results for African Americans should produce effects with the same sign as the categories established by the algorithms (i.e., negative for representation, positive for departures), even though the size of the effect probably differs across racioethnic minority groups.

The classifier algorithms allow for large-sample analyses, but surname is only a proxy for racioethnicity. Surnames usually reflect paternal lineage, so the actual proportion of an individuals’ ancestry can easily differ from the racioethnicity suggested by a surname. However, given that we focus on TMT appointments, individuals’ actual background or how individuals self-identify is less important to the current study than how others (e.g., firm leaders) perceive the individuals. Moreover, misidentification by the classifier should, in general, introduce random error and increase the likelihood of statistically non-significant results. The classifier algorithms rely only on names and have no information on companies or other features associated with individuals. Also, the classifier algorithms examine each name independently, making the explanatory and dependent variables isolated outputs that are free from any internal artifacts that could generate artificial associations. In sum, we can think of no reason why measurement errors would lead to false positive findings.

### Variables and estimation of hypotheses

Our hypotheses deal with the racioethnicity of new appointments as a function of (i) the presence of an individual of a particular racioethnic group on a TMT, (ii) the departure of an individual of a particular racioethnic group from the TMT, and (iii) the CEO’s racioethnicity. Tests of these hypotheses require indicators of the addition of an individual from a specific racioethnic group (i.e., the dependent variable), the presence of an individual from that group on the TMT, the departure of an individual of that racioethnic group from the TMT, and the CEO’s racioethnicity (i.e., the predictors).

Our indicator of an addition is the appearance of a specific individual of a given racioethnicity on a TMT in time t + 1 who does not appear on the TMT in time t. Thus, our dependent variable takes on 7 values, one for each racioethnic minority group (African, East Asian, Hispanic, Indian, Middle Eastern, African American) and one representing the racioethnic majority group. These are not ordered; the values assigned to the racioethnic groups are arbitrary and make no difference in the estimation results except for which category is omitted in the estimation.

For our indicator of the presence of individuals from each racioethnic group, we created dummy variables for each of the racioethnic groups to indicate whether someone from that group was on a particular TMT in year t. For our indicator of CEO racioethnicity, we created dummy variables for each racioethnic group to indicate whether someone from that group was the CEO in year t. Our indicator of departures is whether a specific individual of a given racioethnicity appears on the TMT in time t − 1 but not in time t.

We include indicators of representation, departure, and CEO ethnicity for all the racioethnic minority groups in each estimation. The indicators aligned with a specific outcome test the hypotheses (e.g., Middle Eastern representation, Middle Eastern departure, and Middle Eastern CEO for the appointment of a Middle Eastern individual), and the others constitute controls. Because the classifier provided more specific classifications than just “European” for those with European backgrounds, we also included dummies for representation on the TMT, departure from the TMT, and CEO racioethnicity for each of six European subcategories with >1% representation in the sample (viz., Eastern European, French, Jewish, German, Italian, Nordic). We omit the explanatory variable related to the most common racioethnicity (British) to avoid colinearity. Consequently, our estimation includes a great many controls for representation, departure, and CEO demographics for each company-year. The results are similar with or without using the European subcategories as controls.

In addition to controlling for the presence, absence or CEO for the European ethnicities, our estimation included controls for lags of TMT size, leverage, employment, CAPX/sales, institutional stock ownership, diversity, and industry dynamism. We also controlled for TMT size because increases in TMT size add more opportunities for the addition of individuals from specific groups. We controlled for leverage (debt in current liabilities plus long-term debt divided by total assets) as an indicator of slack because slack also influences risk taking [81]. TMT size was calculated using the Execucomp data that provided the sample of top managers. Employment (divided by 10) and CAPX/Sales came from Compustat. Institutional stock ownership was the fraction of common shares held by institutional investors as reported in the LSEG institutional holdings database (13F).

For our primary measure of firm culture, we used the racioethnic diversity of the TMT. Although there are many dimensions to culture, relative to the racioethnicity of TMT appointments, TMT racioethnic diversity seemed the most relevant. We calculated the index just using underrepresented racioethnic minorities and all others as a single group and using all 13 racioethnicities identified in the data. We calculated Blau’s index [82]:


$$\begin{array}{c} D=\text{1}-\sum_{racioethnicities}{\left(\frac{number\;in\;racioethnicity}{number\;on\;TMT}\right)}^{\text{2}} \end{array}$$

Following Dess and Beard [83], we calculated industry dynamism as follows. First, we aggregated the data to the two-digit SIC code level. Then we regressed industry sales, employment and value added on their lagged values. From the regressions, we divided the root mean squared error by the mean value. Finally, we summed the three measures to give a dynamism indicator.

With an unordered categorical dependent variable, we estimated the model in Stata using the cmp procedure [84] to perform multinomial probit controlling for selection based on an individual being appointed to the TMT in the next period. We used return on assets (ROA) and Tobin’s q in the selection equation; previous analysis indicated they were unassociated with appointees’ racioethnicity.

For each outcome, the explanatory variables included whether each racioethnic group was represented on the TMT (labeled “on”), whether someone from each racioethnic group departed the TMT (labeled “depart”), whether the CEO was from each racioethnic group (labeled “CEO”), and the control variables noted above.

To check the robustness of the estimates we estimated the model multiple ways: multinomial probit, multinomial probit with selection using the cmp program and clustered standard errors, multinomial probit with selection, clustered standard errors and year dummies using the cmp program, and multinomial logit. We also estimated logit models with fixed effects for firm and binary outcomes (appointed someone from a given racioethnic group versus did not appoint someone from that racioethnic group). S1 Appendix presents these estimates. With only one exception, all estimators gave quite similar results. For example, S1 Appendix shows that all three estimators have similar parameters, and the only parameter estimate that is significant in one estimate but not all three is the multinomial probit parameter on having an East Asian on the TMT. The only case where conclusions might differ between estimators is for Indians on the TMT (analysis with year dummies give a different sign on the parameter), yet none of the parameter estimates for Indians on the TMT are statistically significant. Because the large number of parameters created colinearity problems when calculating predicted values with the year dummies, we use the multinomial probit with selection for the predicted values.

With multinomial probit and cmp, estimations can have only one event for each observation. Consequently, when more than one individual joined the TMT in a year, we chose to categorize the arrival of new TMT members for a given year by the least frequent racioethnic group to increase the minimum number of relevant observations for each racioethnic group. Switching the classification rule to use the most frequent racioethnic group gave similar results. The parameters we estimate explain the difference in likelihood for a given category versus a single omitted category (British).

## Results

Table 2 reports firm-year-individual and firm level descriptive statistics. For a correlation matrix, we add dummy variables for the addition of each racioethnic category (any correlation with a multinomial dependent variable would be meaningless because the numeric coding of such variables is arbitrary). Consequently, the correlation matrix is extremely large. Similarly, the full results tables take many pages to present because they involve six outcome variables and 42 parameters in each equation. Therefore, the correlation matrix and full results tables appear in the supplemental information (see S1 and S2 Appendices). The data and syntax are available at https://osf.io/2wgrf/overview?view_only=dc1d4102ddaf41a090cd6d87211df996.

**Table 2:** Descriptive Statistics for Variables in Estimation.

Variable	N	Mean	Std. Error
African On	48,988	0.082	0.274
African Depart	48,988	0.011	0.104
African CEO	48,988	0.013	0.114
East Asian On	48,988	0.096	0.295
East Asian Depart	48,988	0.014	0.119
East Asian CEO	48,988	0.018	0.134
Hispanic On	48,988	0.172	0.377
Hispanic Depart	48,988	0.026	0.158
Hispanic CEO	48,988	0.026	0.158
Indian On	48,988	0.066	0.249
Indian Depart	48,988	0.01	0.101
Indian CEO	48,988	0.015	0.123
Middle Eastern On	48,988	0.051	0.221
Middle Eastern Depart	48,988	0.007	0.081
Middle Eastern CEO	48,988	0.012	0.108
African American On	48,988	0.132	0.338
African American Depart	48,988	0.018	0.132
African American CEO	48,988	0.023	0.149
East Europe On	48,988	0.156	0.363
East Europe Depart	48,988	0.021	0.145
East Europe CEO	48,988	0.027	0.163
French On	48,988	0.202	0.401
French Depart	48,988	0.03	0.169
French CEO	48,988	0.033	0.178
Germanic On	48,988	0.545	0.498
Germanic Depart	48,988	0.091	0.288
Germanic CEO	48,988	0.117	0.321
Italian On	48,988	0.267	0.442
Italian Depart	48,988	0.04	0.196
Italian CEO	48,988	0.049	0.215
Jewish On	48,988	0.332	0.471
Jewish Depart	48,988	0.049	0.216
Jewish CEO	48,988	0.075	0.263
Nordic On	48,988	0.365	0.481
Nordic Depart	48,988	0.055	0.227
Nordic CEO	48,988	0.074	0.262
TMT size t-1	48,988	5.763	1.319
ROA t-1	47,700	0.005	12.46
Q t-1	41,829	1.75	2.354
Employees/10 t-1	48,988	1.787	5.844
Leverage t-1	48,988	0.242	0.408
CAPX/Sales t-1	46,757	0.1	1.365
Diversity t-1	48,988	0.288	0.245
Institutional Ownership t-1	48,988	0.08	0.104
Industry Dynamism t-1	48,988	1.045	3.609

Hypothesis 1 predicts that representation of a racioethnic minority group on the TMT decreases the likelihood of appointing someone else from the same group. Table 3 shows that four of the six relevant parameter estimates are negative and statistically significant. In Table 3, the parameters in the row “Racioethnicity on as Y” indicate the effect of having a member of a racioethnic group on the TMT at time t on the probability of a member of the same racioethnic group joining the TMT at time t + 1. For example, the first entry in the first row (−0.746) is the parameter on the dummy indicating the presence of an African on the TMT at time t in the equation explaining the addition of an African at time t + 1, and the last entry in the first row (−0.666) is the parameter for the presence of an African American in the equation explaining the addition of an African American. The parameter estimates for presence on the TMT (labelled “[Racioethnicity] on as Y”) are negative. Four of the six have *p*-values below .01, with the exceptions being East Asian (*p* = 0.344), and Indian (*p* = 0.834).

**Table 3:** Multinomial Probit Model Parameters for Hypothesized Relationships.

Variable	African	East Asian	Hispanic	Indian	Middle Eastern	African American
Racioethnicity on as Y	−0.746	−0.139	−0.535	−0.0263	−1.575	−0.666
	(0.220)	(0.147)	(0.0972)	(0.125)	(0.379)	(0.141)
	0.000696	0.344	3.72e-08	0.834	3.17e-05	2.13e-06
Depart Racioethnicity as Y	1.496	1.380	1.218	1.317	2.302	1.435
	(0.187)	(0.117)	(0.0878)	(0.126)	(0.244)	(0.131)
	0	0	0	0	0	0
CEO Racioethnicity as Y	1.472	1.327	1.108	0.971	2.342	1.434
	(0.182)	(0.129)	(0.0922)	(0.135)	(0.267)	(0.128)
	0	0	0	0	0	0
TMT size t-1	0.0498	0.0479	0.0491	0.0499	0.0108	0.0552
	(0.0237)	(0.0235)	(0.0168)	(0.0210)	(0.0252)	(0.0190)
	0.0356	0.0417	0.00342	0.0172	0.669	0.00372
Employees/10 t-1	−0.0131	−0.0178	0.000532	0.00626	−0.00169	0.00685
	(0.00839)	(0.00978)	(0.00231)	(0.00176)	(0.00480)	(0.00201)
	0.118	0.0682	0.817	0.000389	0.726	0.000655
Leverage t-1	0.0192	−0.303	0.0189	−0.484	−0.240	0.00757
	(0.0242)	(0.130)	(0.0176)	(0.122)	(0.148)	(0.0372)
	0.426	0.0204	0.283	6.99e-05	0.104	0.839
CAPX/Sales t-1	0.00188	−0.0136	−0.0571	−0.000972	−0.0245	0.000965
	(0.00330)	(0.0619)	(0.0620)	(0.00681)	(0.0695)	(0.00493)
	0.570	0.826	0.357	0.887	0.725	0.845
Diversity t-1	0.328	−0.159	0.0609	0.238	−0.375	−0.231
	(0.298)	(0.302)	(0.207)	(0.236)	(0.366)	(0.214)
	0.270	0.599	0.769	0.314	0.305	0.280
Institutional Ownership t-1	−0.237	−0.320	−0.238	−0.933	−0.122	−0.841
	(0.251)	(0.254)	(0.171)	(0.314)	(0.246)	(0.339)
	0.345	0.209	0.165	0.00295	0.619	0.0130
Industry Dynamism t-1	−0.00602	−0.00307	−0.00344	−0.00190	−0.00368	0.00489
	(0.00598)	(0.00197)	(0.00480)	(0.00404)	(0.00437)	(0.00380)
	0.315	0.118	0.473	0.638	0.400	0.198

*N* = 48,988. Number of firms is 3,807. *P*-values appear below standard errors. *P*-values less than e^-10^ are coded as zero. The reference group is European racioethnicity. “Racioethnicity on” refers to the effect of representation on the TMT; “Depart racioethnicity” refers to the effect of someone with the same racioethnicity exiting; “CEO racioethnicity” refers to the effect of having a CEO with the same racioethnicity. Analyses included dummies for each of the other racioethnicities as controls (i.e., 12 dummies for having someone from that racioethnic group on the TMT, 12 dummies for each racioethnic group departing, and 12 dummies for the CEO’s racioethnicity).

Hypothesis 2 predicts that the departure of someone from a racioethnic minority group increases the likelihood of adding a member of that group. Table 3 shows strong support for Hypothesis 2 across all racioethnic minority groups. All the parameter estimates of departure from the TMT (labelled “Depart [Racioethnicity] on as Y”) are positive, with all *p*-values < .0001.

The results also strongly support Hypothesis 3, which predicts that CEO racioethnicity increases the likelihood of an appointment from the same racioethnic group. Table 3 shows that parameter estimates on CEO racioethnicity associated with appointments in the same racioethnicity are positive for all racioethnic minority groups, with all *p*-values < .0001.

To address whether these statistically significant results also indicate substantively important effects, Table 4 presents predicted probabilities of adding a member of a given racioethnic group. Representation of a racioethnic minority group substantially reduces the likelihood of an appointment of a member of that group. For example, while the base probability of an African appointment is 0.875%, having an African on the TMT drops the probability to 0.191%. For all but Indian and East Asian, the probability of appointing a racioethnic minority group member when the group is not represented on the TMT is two or more times the probability when the group is represented. In short, across four of the six racioethnic minority groups, the probability of adding a racioethnic minority group member is much lower when the group is already represented on the TMT.

**Table 4:** Predicted Probabilities.

Variables	African	East Asian	Hispanic	Indian	Middle Eastern	African American
Racioethnicity on = 0	.00875	.01039	.02282	.00946	.00849	.01539
Racioethnicity on = 1	.00191	.00797	.00894	.00974	.00062	.00451
On=0/On=1	4.578	1.304	2.553	.9714	13.74	3.414
Depart racioethnicity = 0	.00644	.00857	.01687	.00855	.00501	.01101
Depart racioethnicity = 1	.0783	.08566	.1075	.07989	.188	.1071
Depart = 1/Depart = 0	12.16	9.991	6.371	9.343	37.55	9.725
CEO racioethnicity = 0	.00636	.00845	.01716	.00863	.00491	.01093
CEO racioethnicity = 1	.0778	.07496	.09327	.05017	.1912	.1025
CEO = 1/CEO = 0	12.23	8.871	5.435	5.814	38.98	9.378

Racioethnicity on = 0 means the racioethnicity is not on the TMT, racioethnicity on = 1 means the racioethnicity is represented on TMT; Depart racioethnicity = 0 means the person who exited the TMT has a different racioethnicity, depart racioethnicity = 1 means the person who exited the TMT has the same racioethnicity; CEO racioethnicity = 0 means the CEO has a different racioethnicity, CEO racioethnicity = 1 means the CEO has the same racioethnicity.

Also, the departure of someone from a racioethnic minority group substantially increases the likelihood of an appointment of a member of that group. For example, while the base probability of an African appointment is 0.644%, having an African depart the TMT raises the probability to 7.83%. The base probabilities differ across the different comparisons because the predictions hold the other variables in the model at their means. The probabilities for all racioethnic minority groups without a departure range from 0.501% to 1.68% but probabilities with a departure range from 7.8% to 37.55%. In all cases, the probability of adding a member of a racioethnic minority group after someone from that group departs the TMT is more than five times the probability when a member of that racioethnic minority group does not depart. Thus, the departure of a member of a racioethnic group has a much larger impact on appointments than simple representation of that group on the TMT.

Finally, the predicted probabilities indicate the substantive importance of the CEO’s racioethnic group to the likelihood of an appointment of someone from the same racioethnic group. For example, having an African CEO increases the likelihood of an African appointment from 0.636% to 7.78%. The probabilities when the CEO is from the same racioethnic group range from 5.2% to 19.1%, but the probabilities with other racioethnicities range from 0.491% to 1.72%. In all cases, the probability when the CEO is a member of the same racioethnic group is five or more times the probably if the CEO is a member of a different racioethnic group.

Turning to the control variables, effects were sporadic across racioethnic groups (see Table 3). TMT size appears to increase the likelihood of appointments in all the racioethnicities except Middle Eastern, and number of employees may influence appointments of East Asian, Indians and African Americans but no other racioethnic groups. Number of employees associates with increased probability of Eash Asians, Indians, and African Americans. No other firm-level variables had statistically significant parameter estimates for more than two racioethnic groups.

### Changes over time

Descriptively, the proportion of racioethnic minority group members on TMTs in our data increased substantially over time from just over 10% in the early 1990’s to just over 15% in 2014. To test whether the influence of representation, departure, and CEO racioethnicity on appointments changed over time, we compared the strength of each effect before and after the year 2004, the midpoint of our usable time series (see Table 5). Because the cmp and mprobit estimates were quite similar and mprobit provided more robust estimates, our additional investigations used mprobit estimate. Three of the six parameters for representation of a group have larger negative effects in the later data and the two statistically significant differences have one positive and one negative difference at *p* = .10. Five of the six estimates for departure have larger positive effects later in the data (all five significant at *p* < 10). Finally, five of the six parameter estimates for CEO racioethnicity are larger in the later data (three significant at *p* = .10). Thus, if anything, the pattern of results appears to be strengthening over time.

**Table 5:** Comparing Parameters for Representation, Departures, and CEO Racioethnicity across Time.

Variables	African	East Asian	Hispanic	Indian	Middle Eastern	African American
On early	−0.5705	−0.2209	−0.5394	0.4453	−4.536	−0.7442
On late	−1.154	−0.1279	−0.6989	−0.08503	−1.462	−0.8618
*χ* ^2^	1.68	0.1175	0.6881	3.634	5.851	0.1515
*p*-value	0.1949	0.7318	0.4068	0.0566	0.01557	0.6971
Depart early	1.086	0.8141	0.8005	0.4277	3.431	1.191
Depart late	2.062	1.827	1.618	1.661	2.42	1.756
*χ* ^2^	5.49	10.38	14.9	9.514	1.821	3.751
*p*-value	0.01912	0.00128	0.00011	0.00204	0.1771	0.05278
CEO early	1.342	1.05	0.9133	0.5202	3.644	1.433
CEO late	1.851	1.537	1.442	1.15	2.451	1.608
*χ* ^2^	1.46	2.64	6.001	3.306	3.494	0.3438
*p*-value	0.227	0.1042	0.0143	0.06903	0.06158	0.5576

“Early” parameters estimated on 1992–2004 data. “Late” parameters estimated on 2005–2019 data. “On” refers to the effect of representation on the TMT; “Depart” refers to the effect of someone with the same racioethnicity exiting; “CEO” refers to the effect of having a CEO with the same racioethnicity.

### The absence of spillover effects

Our analysis of spillover effects depends on the complete results presented in S2 Appendix. If firm leaders treat minority groups as a general category, we would expect the representation or departure of any racioethnic minority group member to influence appointments in that group and the other racioethnic minority groups. We observe little evidence of this. Instead, the effects are isolated largely to one racioethnic group. Only 10 of the 90 parameters on cross-racioethnic influences for the appointment of racioethnic minority groups are statistically significant (*p* < .10) which is not far from what we would expect by chance. Moreover, the parameters that are statistically significant have little apparent pattern. In short, the results indicate that firm leaders differentiate among racioethnic minority groups. These results highlight the utility of examining specific racioethnic groups rather than the broader categories often used in conversations about racioethnicity.

Finding that the effects of representation, departures, and CEO racioethnicity are largely isolated within racioethnic minority groups also rules out one possible explanation for our results—that representation of one racioethnic minority group decreases the likelihood of appointments from any other racioethnic minority group. If higher-level categorizations (e.g., Asian versus East Asian and Indian) determined our results, we should see many substantial parameters across racioethnic groups. We do not. Put simply, the effects of TMT members’ racioethnicity on TMT appointments are specific to that racioethnicity; there are minimal spillover effects of consequence.

## Additional analyses

### Robustness

We conducted several multinomial logit analyses to assess the robustness of our results. We used multinomial logit because it can handle fixed effects, whereas multinomial probit only handles random effects. First, we set up separate binary variables for appointments of individuals from each racioethnic minority group versus appointments from any other racioethnic group. Then, we estimated separate logit estimates with fixed effects for firm, modeling the likelihood of appointing someone from that racioethnicity versus any other racioethnicity. A series of conditional logits is very similar to a multinomial logit estimate; they use precisely the same numerator in the model but can include fixed effects for firm. These estimates also include all additions, not just one addition per firm per year and allow fixed effects for firm. The results align closely with the multinomial probit results.

As noted above, a conventional multinomial logit without endogeneity controls gave similar results. We considered a fixed effects multinomial logit, but this approach is computationally infeasible with many categories and a large sample [85,86]. Instead, we estimated partial models (e.g., using four of the outcome categories instead of the full six). The results of the partial models remain consistent with the multinomial logit results.

Because the multinomial logit only allows one outcome for each observation, we had chosen to include the least common outcome. We also reversed the order using the most common racioethnic group, but it did not substantively change the results (see S3 Appendix).

We also address questions about the reliability of the racioethnicity classifier by replicating our analysis using a set of racioethnic group data produced by an independently developed algorithm, the one described in Ambekar et al. [73]. The results are extremely similar between the two classifiers.

Because our definition of TMT depends on employee compensation, we also check whether the same individual might be on a TMT in year t–1, drop from the reported team in year t, and then reappear in t + 1. Results do not change when counting arrivals only when someone was not on a TMT in t − 1, t − 2, and t − 3.

### TMT diversity

Our analysis so far looked at direct associations between racioethnic groups and appointments. We also explored whether the racioethnic diversity of the TMT in t-1 moderated the effects. We measured diversity by the Blau heterogeneity index 1 - Σp_i_^2^ where p_i_ is the proportion of the TMT in the i^th^ racioethnicity [87]. We focus on the interactions of theoretical interest—the three African variables in the African equation, the three East Asian variables in the East Asian equation, etc. The test that the estimated interaction parameters equal zero was statistically significant, χ^2^(18) = 166.7, *p* < .0001. The interactions of TMT diversity in t-1 with the variables showing representation on the TMT all have positive parameters, and the interactions with departures and CEO racioethnicity all have negative parameters. All but three of the 18 parameters are statistically significant at *p* < .01 and the remaining three at *p* < .10. The interactions reduce the magnitude of the main effects, but at the mean of diversity, the total effects have the same signs and remain statistically significant. At the maximum value of diversity, TMT diversity does eliminate the statistically significant effect and even reverses the effect for some of the relevant parameters. In summary, TMT diversity reduces the effects found above but only eliminates them for some racioethnicities and only at the highest level of diversity.

### Amount of racioethnic minority group representation

The analyses above assume that representation on the TMT is all that matters; they have not examined whether the effects change as amount of representation increases above one. We addressed this question by estimating a model that includes the number and number squared from each racioethnic minority group (see S4 Appendix).

Using linear and squared terms on number of representatives from a racioethnic group, we find four of the six linear terms have statistically significant negative parameter estimates and four of the six squared terms have statistically significant positive parameter estimates (see S4 Appendix). However, in all cases, the parameter on the linear term is substantially larger than the parameter on the squared term. Thus, although the effects appear weaker when more members of a racioethnic minority group are on the TMT, it would take many (four or more) on the TMT to make any of the net effects positive.

### CEO racioethnicity

We also explored the possibility that CEO racioethnicity moderates the effects. Due to a lack of observations (many combinations of CEO racioethnicity and racioethnicity of those on the TMT were not in the data), we could not interact all the CEO racioethnicities. Instead, we created a dummy variable that represented when the CEO was non-European (i.e., a racioethnic minority group member) and entered it as a main effect and interacted it with the representation and departure explanatory variables. All six interactions of the CEO dummy variable and racioethnic minority group representation on the TMT were negative and statistically significant (p < 0.01); the negative association between racioethnic minority representation on the TMT and subsequent appointments to the TMT is stronger when the CEO has a non-European racioethnicity. Three of the six interactions of the CEO dummy variable and the departure of racioethnic minority group members were positive and statistically significant (p < 0.01), indicating that the positive association between departures of racioethnic minority group members from the TMT and the appointment of members from the same racioethnic minority group as the person who left is stronger when the CEO has a non-European racioethnicity. A joint test that the parameters on the interactions equaled zero was statistically significant, χ^2^(11) = 274.5, *p* < 0.001. In sum, having a non-European CEO appears to strengthen the representation and at least some of the departure effects we observed in our main analyses.

### Gender

Prior research investigating the appointment of women to TMTs has not considered whether gender and racioethnicity interact. We tested a model with number of women on the TMT entered both as a main effect and as an interaction with all the right-hand side variables. Few of the interactions were statistically significant. A joint test that the theoretically relevant parameters differ from zero was statistically significant, χ^2^(18) = 18.2, *p* = 0.44. However, looking at the individual parameters, only two were statistically significant and only at the *p* < 0.10 level. Overall, the evidence does not seem to support a conclusion that the number of women on the TMT moderates the effects described by the Hypotheses.

## Discussion

We sought to better understand why TMTs remain much less racioethnically diverse than employees at other levels and the general population, and our results provide robust evidence that the existing racioethnic composition of the TMT predicts the likelihood of subsequent appointments of racioethnic minority group members to the TMT. Representation of a racioethnic minority group on a TMT was associated with a decrease in the likelihood of appointing another member of that group to the TMT. Also, the departure of a racioethnic minority group member from the TMT was associated with an increase in the likelihood of an appointment of a member of that group. Together, these results suggest that firm leaders avoid increasing or decreasing representation of a racioethnic minority group on the TMT, and evidence that resistance to changes in either direction within a dimension is commonly interpreted in the literature on decision-making as evidence of status quo bias (i.e., a reversal test; see [23]).

We also observed that CEO racioethnicity represents an important counterpoint to the effects described above. Although racioethnic minority groups were underrepresented among CEOs in our sample, we found that having a CEO who was a racioethnic minority group member was associated with an increased likelihood of appointments of members of the CEO’s racioethnic group to the TMT. We anticipated this effect because CEOs have considerable power in TMT appointment decisions—certainly more power than other TMT members (e.g., [26])—and CEOs who are racioethnic minority group members likely experience intergroup dynamics differently than racioethnic majority group members. Overall, the results clearly show that existing racioethnic representation on the TMT affects subsequent TMT appointments, and the pattern suggests that intergroup dynamics are a contributing factor, above and beyond the role of stereotypes.

Before moving on, it is important to note that although the results were remarkably consistent across racioethnic groups and analyses, there was some variability. In particular, the effects of having East Asians and Indians on the TMT did not significantly reduce future TMT appointments of East Asians and Indians, respectively, even though the parameter estimates for all racioethnic minority groups, including East Asians and Indians were negative. The current data cannot address the reason for this variability, but it could be associated with how Asian Americans achieve higher levels of education, earn higher incomes, and report less (but still considerable) discrimination than other racioethnic minority groups in the United States [88,89]. As indicated above, this interpretation is speculative, but examining this result could be a worthwhile direction for future research. In any case, the results underscore how each social group has a unique history and set of contemporary challenges that may influence the way firm leaders and the general public conceptualize diversity and respond to issues involving a particular group [45]. Even though researchers commonly aggregate across racioethnic minority groups, numerical minority status in a given context is just one of potentially many characteristics of any group (and not necessarily the most distinctive one). Therefore, future research should continue to test for and seek to understand differences across racioethnic minority groups.

### Status uncertainty

Given that the current paper is the first to theorize a role for status uncertainty as a factor that influences TMT appointments, it is important to evaluate the evidence for our theory. Both stereotypes and status uncertainty may underpin some effects we observed. However, status uncertainty can explain some observations that stereotypes cannot.

Our theory asserts that two different types of status concerns influence TMT appointments. First, firm leaders have concerns about preserving their status and control within the TMT. This *intragroup* status concern makes firm leaders reluctant to appoint additional members of the same racioethnic group, fearing coalitions may form among individuals from the same racioethnic group [60,61]. Second, firm leaders may worry about their TMT losing status relative to other TMTs if it includes members from low status racioethnic groups. This *intergroup* status concern may be especially acute when considering the prospect of appointing a second individual from a particular stigmatized group to the TMT; having multiple individuals from a group is more likely to influence how people think about the TMT (i.e., more likely to impact the group prototype; [50]). Taken together, our theory of intra- and intergroup status concerns provides a novel explanation for facets of the effects we observe.

The stereotyping account asserts that stigma against racioethnic minority groups negatively influences firm leaders’ perceptions of TMT candidates from those groups, which inhibits their appointment to the TMT. Thus, the stereotyping account operates at the level of individual candidate evaluations; it does not explain why having one person from the group would negatively influence adding another. If anything, having someone from a stigmatized group on the TMT who performed well (or even adequately) should provide salient counterexamples that help break down stereotypes [32,52,90]. Consistent with this idea, our additional analyses show that as the number of members of a racioethnic minority group increases, the effects we observe begin to weaken somewhat. Thus, stereotypes and status concerns inhibit appointments of racioethnic minority group members to the TMT, whereas repeated interactions with racioethnic minority group members appears to moderate but not eliminate these effects. In any case, stereotypes cannot explain all our results, which indicates the utility of considering the role of status concerns.

The presence of the effects of CEO racioethnic minority group membership predicted by Hypothesis 3 and the absence of interactions of CEO racioethnicity with racioethnic representation on the TMT and with the racioethnicity of departures from the TMT (reported in the additional analyses above) also are consistent with our status uncertainty account. Our rationale for the main effects of CEO racioethnicity on TMT appointments predicted by Hypothesis 3 included the argument that CEOs who are members of a minority group should experience less status uncertainty from the prospect of adding a member of their own group [44]. Therefore, we expected CEOs who were racioethnic minority group members to be less invested in the status quo when doing so would involve limiting opportunities for members of their own racioethnic group. However, social identity theory predicts that all CEOs—irrespective of whether they are members of a racioethnic minority group or the majority group—should experience status uncertainty when considering the prospect of appointing someone to the TMT who is a member of a racioethnic group other than their own [8,24]. Thus, we would not expect to observe interactions of CEO racioethnicity with racioethnic representation on the TMT and with the racioethnicity of departures from the TMT. Although one must always be cautious when interpreting null findings, the combined pattern of results is consistent with status uncertainty.

Before turning to potential alternative explanations for the results we observed, we note that negative stereotypes and status concerns are fundamentally related, even though they have distinguishable effects. As mentioned above, the prospect of any new TMT appointment can trigger status concerns [8,24]. However, negative stereotypes exacerbate status concerns because firm leaders may consciously or unconsciously experience more uncertainty and be more concerned about the potential disruption to within- and across-group status hierarchies when considering the appointment of someone who is a member of a negatively stereotyped group. In other words, negative stereotypes operating at the intrapsychic level underpin and aggravate status concerns operating at the intergroup level.

### Alternative perspectives

Taken together, our results align more with our account than alternatives. One alternative account is that our results could reflect a genuine, principled commitment to increasing racioethnic diversity. One might argue that if firm leaders sought to optimize racioethnic variety (i.e., heterogeneity; [66]), they would include only one TMT member from each racioethnic group, leaving room for members from other groups. This account makes sense if the level of variety were already high. However, with low to moderate levels of variety, as in our data set, adding a second member from a given minority category can have a positive effect on variety [65]. In other words, the account based on a principled commitment to diversity ignores the reality that the vast majority of TMT members in our sample have European lineages (Table 1; see also [5]). Considering the current demographics of TMTs, having multiple members from the same racioethnic minority group is much less of a barrier to increasing variety than having a disproportionate number of whites. Thus, the principled account seems unlikely.

Another alternative account worth considering is the possibility that the results reflect efforts to maximize expertise on the TMT. This account assumes that TMT members’ racioethnicity associates with cultural knowledge that benefits the firm [90–92]. From this perspective, the first TMT member from a given racioethnic group provides additional expertise but appointing additional members from the same racioethnic group offers diminishing returns and introduces less novel expertise than adding someone from a different racioethnic group. Thus, the expertise perspective appears to account for both the effect of group representation on appointments and the effect of departures on replacements.

However, the expertise account has several limitations. First, for the expertise account to explain our results, one must assume that firms generally seek to increase the breadth of cultural expertise on the TMT. Instead, it seems more likely that firms would concentrate on the type of expertise most relevant to their businesses. Consequently, we should observe positive rather than negative effects of group representation on appointments. Second, the positive association between CEO racioethnicity and adding another individual from the same racioethnic group contradicts the expertise account, unless CEOs seek to deepen the cultural expertise they already bring to the TMT (which takes us back to the first problem noted above). Third, the expertise account assumes that firms must appoint individuals to the TMT to obtain expertise associated with racioethnicity. TMTs routinely rely on both other employees in the firm and on outside consultants to provide expertise in domains outside the TMT’s range of expertise. Taken together, the limitations of the expertise account prompt us to favor our account.

The results also might reflect TMT networks. In this explanation, the departure effects predicted by Hypothesis 2 may reflect a departing member of the TMT recommending people from their network to fill the position, and their networks are likely to include people from their own racioethnic group [66]. Likewise, CEOs’ networks also are likely to include members of their own racioethnic group, which may contribute to the positive association between CEO racioethnicity and TMT appointments of individuals from the same racioethnic group.

We cannot rule out the possibility that network effects operate alongside the social identity processes articulated by our theory, but a networks account struggles to explain all the findings. Most important, the results that support Hypothesis 1 indicate that social identity effects dominate networks effects. From a networks perspective, having a racioethnic minority group represented on the TMT should increase the availability of candidates from the same racioethnic group and increase the likelihood that a second member of the racioethnic group be appointed to the TMT. However, we observe the exact opposite; having a racioethnic minority group represented on the TMT decreases the likelihood that a second member of the racioethnic group is appointed to the TMT. In short, the results are consistent with our predictions based on social identity theory and inconsistent with a networks account.

We wish to note that our data cannot differentiate between conscious and unconscious racioethnic bias. Firm leaders may make appointment decisions that indicate a clear preference for the status quo without being conscious that racioethnic similarity or dissimilarity influences that preference. Psychological research shows that unconscious associations can influence behavior (i.e., associations individuals hold without being aware of them), even if individuals subscribe to principles that run counter to them [93]. Firm leaders may attribute their candidate preference to apparent features of the candidate (e.g., someone they can trust, someone who shares their vision and values) but be unaware that racioethnic similarity or dissimilarity influences their perceptions and attributions [94,95]. In short, our theory and our data are agnostic about the extent to which the effects we observe are deliberate.

### Theoretical implications

This paper contributes to the literatures on TMT appointments by being the first to examine TMT racioethnicity. Complementing prior research that examines other types of diversity (e.g., age, functional experience, education, gender), our results suggest that the existing racioethnic composition of a TMT is significantly associated with who will join the team. However, our results contrast with research on racioethnicity at other levels of firms that show effects in the opposite direction. Specifically, research on organizational and relational demography finds positive effects of demographic representation on subsequent additions to teams; people are more likely to join and remain in organizations that include people who are demographically similar to themselves [95–97, see also 53]. Also, hiring managers tend to favor applicants who seem to fit with the organization, and fit depends in part on perceived similarity [51,95,98,99]. In short, prior research indicates that racioethnic group representation at other levels of firms facilitates entries of individuals from the same racioethnic group because it influences both existing and prospective group members. In contrast, our results indicate that racioethnic group representation on TMTs is negatively associated with appointments of additional members of the same racioethnic minority group.

We also extend theories of the antecedents of TMT appointments by articulating how status uncertainty inhibits TMT appointments of racioethnic minority group members. We theorize that firm leaders experience uncertainty and concern about how new appointees could affect the dynamic of the TMT and the status of the TMT relative to other TMTs. Whereas prior research focuses on the diversity-inhibiting effects of stereotypes, our status uncertainty account addresses intergroup dynamics that operate in parallel with stereotypes. Thus, our research helps create a more comprehensive theory of the antecedents of TMT diversity.

Examining antecedents of racioethnicity diversity on TMTs is intrinsically valuable, but it also provides an opportunity to examine the social dynamics of diversity when multiple categories exist within a salient demographic dimension (in contrast to research on gender that involves a majority group and only one minority group). By examining racioethnicity, we can explore whether the effects of group representation are specific to one group or spill over to multiple groups. We also can assess the granularity of the categories people use when considering racioethnicity. Importantly, we find highly specific effects of racioethnicity with high granularity: Having a member of a given racioethnic group on the TMT, depart from the TMT, or as CEO is almost exclusively associated with additions from the same racioethnic group with few spillovers to other groups. That is, the results strongly suggest that people categorize members of racioethnic minority groups not as “minorities” or “people of color” but as members of more specific racioethnic groups. Moreover, people readily differentiate among groups that often are subsumed under the label “Asian.” Of course, our surname-based classifier may not capture all the meaningful categories people use when thinking about racioethnicity, and the relevant categorizations undoubtedly vary across national contexts. Nevertheless, our findings indicate that future research should measure racioethnic group membership at a finer grained level than commonly used.

We also note that mechanisms and outcomes probably differ across types of diversity. For example, adding a white woman to a TMT may influence subsequent TMT appointments and TMT behavior differently than adding a Hispanic or African American man. The conscious or unconscious factors that dominant group members (i.e., white males) associate with white women differ from the factors they associate with, for example, Hispanic or African American males. Similarly, diversity in other demographic categories (e.g., functional experience) may operate differently than gender or racioethnicity. In short, different dimensions of diversity may influence outcomes differently, and a given dimension of diversity may not have the same influence across all outcomes. For example, early work on opinion diversity in executive decision making found that opinion diversity improved decision quality but reduced commitment to implementing decisions [100,101]. If anything, it would be more surprising if all kinds of diversity had the same influence on a wide range of outcomes than if their influences vary across types of diversity and outcomes.

An additional advantage of the current study also is that it examines the addition of individuals from specific racioethnic groups, which directly aligns with the decisions managers actually make. Decision makers select individuals to join TMTs, not the aggregate team composition commonly examined in diversity research. Our longitudinal approach offers added precision to the cross-sectional approach and allows for controls for stable firm-specific differences (via fixed effects). Consequently, it complements traditional methods.

### Practical implications

Firm leaders face pressure to appoint more racioethnic minority group members to the TMT for both ethical and instrumental reasons. Inhibiting participation in firm leadership (and in the workforce in general) on the basis of racioethnic group membership is unethical from numerous perspectives (e.g., [102]), and perceptions that others are acting unethically often fuels activism and protest [103]. Although legal and regulatory pressure from the US federal government has decreased recently, other important stakeholder groups continue to scrutinize diversity, including some employees, consumers, institutional investors, US state governments, and European regulators that have jurisdiction over multinational firms who conduct business in the zone (e.g., [104–106]). Moreover, shareholders should note evidence that demographic diversity can, at least in some contexts, increase firm performance [6,47,48]. In contrast, there is little, if any, evidence that homogeneity increases firm performance, which raises questions about why accepting a homogenous TMT should be an acceptable default in a diversifying society [107]. In sum, people internal and external to the firm should care whether senior leadership reflects open access to opportunity.

The current results help to explain why TMT diversity changes slowly despite attention from multiple stakeholders for multiple reasons and provide guidance about when and how firm leaders should respond. The results suggest that status quo bias plays a role in TMT appointments and inhibits full use of available executive talent. Specifically, the representation effect indicates that firms with racioethnic minority group members on the TMT are less likely to appoint candidates from groups already represented on the TMT, and this effect may reflect a tendency for firm leaders to overlook the most qualified individual due to their racioethnic background rather than appoint them based on their abilities. Moreover, the departure effect indicates that when racioethnic minority group members depart the TMT, firms are more likely to appoint candidates from the same racioethnic minority group as the person who left. Again, the departure effect may reflect a tendency for firm leaders to overlook the most qualified individual, but in this case firm leaders may do so based on (conscious or unconscious) motivation to replace the “missing” racioethnic group rather than appoint the best candidates based on their abilities. In either case, the results identify instances when appointments may be suboptimal, and firm leaders should take extra care to evaluate how the racioethnic status quo of the TMT may be influencing their appointment decisions.

The results also indicate that the strength of the representation and departure effects tend to decline somewhat as racioethnic diversity of the TMT increases, which suggests that firms with more homogeneous TMTs should pay particular attention to how racioethnicity may be biasing appointment decisions. That said, the representation effect indicates that firm leaders should not assume that a single appointment of a racioethnic minority group member will immediately eliminate racioethnic bias in appointment decisions. Instead, representation may intensify bias associated with the represented group that would only decline if diversity (in aggregate) substantively increases. In any case, being aware of the potential for bias is an important precondition to leveraging strategies that can reduce bias [107,108].

### Limitations

As with any research, this paper has limitations. First, it is important to acknowledge that our longitudinal analyses produce conditional correlations and do not provide direct evidence of causality. Although we address some concerns with our robustness checks that included a variety of models and controls, these analyses are vulnerable to problems related to incidental parameters, unobserved confounders, and violations of strict exogeneity. In short, the pattern of results is consistent with our theory, but our causal inferences are unsupported by the analyses.

Also, our theory is about firm leaders’ appointment decisions, but our analyses do not directly examine key aspects of our theory, including board composition and board-level dynamics. Thus, the proposed mechanism is inferred rather than directly observed. We therefore cannot completely rule out alternative explanations such as homophily, network-based recruitment, sponsorship dynamics, or broader organizational inertia.

Another limitation is that our study defines TMTs using the ExecuComp data on top management compensation. Other definitions of TMTs might yield different results [109]. However, our definition of TMTs has been widely used in the literature [20,68–72], and the highest paid individuals are normally part of the TMT. The individuals we identify as TMT members would normally also be on the TMT using other definitions. Likewise, our measure may not identify some departures from the TMT that would be included under a more expansive definition of TMT, but the departures identified by our definition would be departures under a more expansive definition of TMT. In short, our definition is potentially restrictive but unlikely to include individuals that other definitions would exclude. Thus, the effects we observe are unlikely to be an artifact of extraneous individuals in the sample.

Furthermore, our study uses surnames to identify racioethnicity, and surname identification is sure to include measurement error. Furthermore, our surname-based classifier would misclassify African Americans who have European surnames. Although we sought to identify as many African Americans as possible, we probably did not identify all of them, resulting in some misclassifications. Moreover, some of our racioethnic categories are broader than others (e.g., East Asian versus Indian) and differ in terms of heterogeneity within categories. However, it is difficult to understand how any of these potential limitations could explain our results. In general, inaccuracies in identifying racioethnicity would weaken, not strengthen the observed effects. Moreover, the reversal in sign of the CEO and departure effects relative to the group representation indicates that our classification scheme is not for some unknown reason producing results that mistakenly indicate wholesale trends in any one direction.

Finally, our results only address the dynamics associated with racioethnicity in the United States during recent years. The salience and importance of various racioethnic distinctions undoubtedly differ across countries and time. That said, the social identity processes—status uncertainty and stereotyping—underpinning our results may generalize, even though the specific ways that people parse their social world into groups depends on context.

## Conclusion

Many firms express interest in increasing diversity across levels of the organization, including leadership positions, but TMTs remain considerably less diverse than other employees and the general population. Our results complement and extend the prior research on TMT appointments by documenting that racioethnic minority group representation on a TMT predicts the likelihood of appointing someone from the same group to the TMT. Coupled with finding that the racioethnicity of those departing TMTs and a CEO’s racioethnicity also predict the likelihood of appointments to TMTs, our results suggest that status uncertainty partially accounts for why TMT racioethnic diversity lags.

## Supporting information

S1 Appendix Correlation Matrix. (XLSX)

S2 Appendix Full Estimation Results. (XLSX)

S3 Appendix Reversed Allocation of Arrival When More Than One Arrival. (XLSX)

S4 Appendix Linear and Squared Effects when More Than One in a Racioethnic Group. (XLSX)
