# Supplementary material for: Existing racial and ethnic representation on top management teams predicts subsequent top management team appointments that preserve the demographic status quo
Source: PLoS One. 2026 Sep 8;21(9):e0357710. doi: 10.1371/journal.pone.0357710 (PMC13552966; doi:10.1371/journal.pone.0357710)
Supplement: Supplementary file 1 [file pone.0357710.s006.docx]

PONE-D-24-54283

Existing racial and ethnic representation on top management teams influences subsequent top management team appointments in ways that preserve the demographic status quo

Dear Woojin Yoon:

Thank you very much for the opportunity to revise our manuscript. I apologize for the additional time it took us to submit the revision, but two of us have been dealing with some major health issues. However, we now have carefully engaged with all the feedback, and we believe that the paper is much stronger as a result. We hope you agree.

We address each point raised by you and the reviewer below:

**Journal Requirements:**

*1. Please ensure that your manuscript meets PLOS ONE's style requirements, including those for file naming.*

We carefully formatted all materials. Please accept our apologies for failing to conform to the journal’s standards in our initial submission.

*2. Thank you for uploading your study's underlying data set. Unfortunately, the repository you have noted in your Data Availability statement does not qualify as an acceptable data repository according to PLOS's standards.*

We uploaded our data to OSF: https://osf.io/2wgrf/overview?view_only=dc1d4102ddaf41a090cd6d87211df996

*3. In the online submission form, you indicated that your data will be submitted to a repository upon acceptance.  We strongly recommend all authors deposit their data before acceptance, as the process can be lengthy and hold up publication timelines. Please note that, though access restrictions are acceptable now, your entire minimal dataset will need to be made freely accessible if your manuscript is accepted for publication. This policy applies to all data except where public deposition would breach compliance with the protocol approved by your research ethics board. If you are unable to adhere to our open data policy, please kindly revise your statement to explain your reasoning and we will seek the editor's input on an exemption.*

We uploaded our data to OSF:

https://osf.io/2wgrf/overview?view_only=dc1d4102ddaf41a090cd6d87211df996

*4. When completing the data availability statement of the submission form, you indicated that you will make your data available on acceptance. We strongly recommend all authors decide on a data sharing plan before acceptance, as the process can be lengthy and hold up publication timelines. Please note that, though access restrictions are acceptable now, your entire data will need to be made freely accessible if your manuscript is accepted for publication. This policy applies to all data except where public deposition would breach compliance with the protocol approved by your research ethics board. If you are unable to adhere to our open data policy, please kindly revise your statement to explain your reasoning and we will seek the editor's input on an exemption. Please be assured that, once you have provided your new statement, the assessment of your exemption will not hold up the peer review process.*

We uploaded our data to OSF:

https://osf.io/2wgrf/overview?view_only=dc1d4102ddaf41a090cd6d87211df996

*5. Please include your full ethics statement in the ‘Methods’ section of your manuscript file. In your statement, please include the full name of the IRB or ethics committee who approved or waived your study, as well as whether or not you obtained informed written or verbal consent. If consent was waived for your study, please include this information in your statement as well.*

On p. 15 of the manuscript, we now make the following declaration:

The Institutional Review Board at the University of California, Irvine does not require and will not provide approval for this research because it involves secondary uses of identifiable private information that is publicly available and external of the University (Exempt Category 4). For the same reason, informed consent was not required.

*6. We notice that your supplementary tables are included in the manuscript file. Please remove them and upload them with the file type 'Supporting Information'. Please ensure that each Supporting Information file has a legend listed in the manuscript after the references list.*

We submitted the supplementary tables in the appropriate format and include a legend list in the manuscript after the references list.

***Reviewer #1:*** *This paper investigated whether existing racial/ethnic composition of TMT influences later appointment of TMT members. The authors shed light on status concerns to induce the tendency of appointment decisions. This paper has significant potential. At the same time, I have some concerns about both theory and methods; I hope my comments will be useful to authors to push the manuscript forward.

1. From a theoretical standpoint, it is not well explained why CEO and other TMT members show different patterns. Like CEO, other TMT members, especially those in minority groups, may want to bring in more minorities whether it is because they feel emotionally connected or because they need more political capital. Thus, by extension, the authors need to explain why individuals care more about the group-level status quo than their personal preferences. Along similar lines, it seems less convincing that the current explanation assumes that TMT members want to preserve status quo and that it is the best thing to do. Although the authors describe this as status quo bias, but it needs to be explained further.*

In the prior draft of the paper, we used the term “firm leaders” when referring to the agents who make TMT appointments, and our failure to define this term left open opportunities for misunderstanding. We now explicitly state on p. 3 that Boards of Directors have the formal power to make TMT appointments, although other parties may influence the decision (e.g., CEOs, large investors). We hope this description of the corporate governance structure eliminates confusion about who makes TMT appointments. This governance structure is important because Boards of Directors of companies in our sample were overwhelmingly white; the 2016 Board Diversity Census found that 87.2% of board members of Fortune 500 companies were white (Alliance for Board Diversity, Deloitte). Thus, it is the overwhelmingly white Board of Directors that is invested in the group-level status quo (not the CEO or other TMT members).

Our results indicate that firm leaders—especially the Board of Directors—is invested in the racioethnic status quo because they resist adding additional members of a racioethnic minority group that is already represented on the TMT and they also are much more likely to appoint a racioethnic minority group member when a member of the same group recently departed the TMT.

Our results also suggest that CEO racioethnicity has a different influence on appointment decisions than the overall racioethnic composition of the TMT, and we provide on p. 14 three reasons why this may be the case. In short, we argue that the effect of CEO racioethnicity differs because (1) CEOs who are racioethnic minority group members are in a strong position to advocate for members of their own racioethnic minority group to join the TMT, (2) CEOs who are racioethnic minority group members should be less invested in limiting opportunities for members of their own racioethnicity group, and (3) candidates from the same racioethnic minority group as the CEO may be more attracted to the role.

*2. The front part of the current manuscript does not appear to guide the audiences to understand the research question and why it is important. I suggest for the authors to develop a clear research question which the authors can use to guide the research.*

We made several revisions to the introduction to be more explicit about our research question and how it guided our research. For example, we now begin the second paragraph with the following: “We examine how the existing racioethnic composition of TMTs influences subsequent appointments of racioethnic minority group members to TMTs to shed light on why TMTs remain relatively homogeneous in terms of racioethnicity despite widespread institutional pressure to increase diversity (e.g., Hunt et al. 2015; Richard, Triana, & Li 2021; Ely & Thomas 2020).” We then contrast our approach and our theory with prior research on the topic, briefly review our results, and finish by identifying four main contributions to the literature.

*3. The efforts and methods to identify the racioethnicity of the TMT members are vey impressive, but there is very limited information about the firms to which the TMT belongs, other than ROA and Q. For example, firm-level covariates such as ownership structure, strategic priorities, corporate culture can have a very significant impact on TMT appointments and should be controlled for. Similarly, industry-level information such as industry dynamics and market conditions need to be controlled for.*

We appreciate the suggestion given that the ExecuComp database is large and includes all S&P 1500 firms plus companies that were once part of the 1500 index and are still trading. We added several firm-level covariates to our analyses, including TMT size, firm size (# of employees), leverage, CAPX/sales, diversity of the TMT (as an index of corporate culture), institutional ownership, and industry dynamism. Few of these factors had any significant effect and none had systematic effects for any racioethnic group. In short, our results remain effectively the same with the additional controls included in the analyses.

*4. As the authors note, the detailed classification of racioethnicity is a huge strength of the paper. However, the interpretation is lacking in the explanation of each racioethnicity individually. For example, there needs to be more explanation as to why East Asians and Indians do not show similar patterns to other minority groups.*

Unfortunately, our data cannot speak to why representation of East Asians and Indians had less negative effects on subsequent TMT appointments than representation of other racioethnic minority groups, but we agree that it was a mistake not to address these results directly in the discussion. We added the following the pp. 34-35:

Before moving on, it is important to note that although the results were remarkably consistent across racioethnic groups and analyses, there was some variability. In particular, the effects of having East Asians and Indians on the TMT did not significantly reduce future TMT appointments of East Asians and Indians, respectively, even though the parameter estimates for all racioethnic minority groups, including East Asians and Indians were negative. The current data cannot address the reason for this variability, but it could be associated with how Asian Americans achieve higher levels of education, earn higher incomes, and report less (but still considerable) discrimination than other racioethnic minority groups in the United States (Hirshman & Wong, 1986; McMurtry et al. 2019). In any case, these results underscore how each social group has a unique history and set of contemporary challenges that may influence the way firm leaders and the general public conceptualize diversity and respond to issues involving a particular group (Sidanius & Pratto, 1999). Even though researchers commonly aggregate across racioethnic minority groups, numerical minority status in a given context is just one of potentially many characteristics of any group (and not necessarily the most distinctive one). Therefore, future research should continue to test for and seek to understand differences across racioethnic minority groups.
