# Supplementary material for: Existing racial and ethnic representation on top management teams predicts subsequent top management team appointments that preserve the demographic status quo
Source: PLoS One. 2026 Sep 8;21(9):e0357710. doi: 10.1371/journal.pone.0357710 (PMC13552966; doi:10.1371/journal.pone.0357710)
Supplement: Supplementary file 2 [file pone.0357710.s007.docx]

PONE-D-24-54283

Existing racial and ethnic representation on top management teams predicts subsequent top management team appointments that preserve the demographic status quo

Dear Soo Keong Yong:

Thank you very much for the opportunity to revise our manuscript. We carefully engaged with the reviewer’s comments, and we believe that the paper is clearer as a result. We hope you agree.

Editor Comments:

*(1) The data description is not sufficiently transparent, specifically how the data are being cleaned and used in the regression analysis, is not clear. For example, the paper did not describe the total number of unique firms in the sample (it only reports person-firm-years), and it does not describe how ExecuComp defines "top managers". In addition, there is no description of how missing data and sample attrition – which is common in micro firm-level data – is being managed and documented.*

Thank you for identifying ways we could improve transparency in the our description of our data.

We now report the number of unique firms in the sample in the abstract, main text on p. 4, and note under Table 3 on p. 26.

We now explain on p. 16 in the data construction section of the manuscript how ExecuComp defines “top managers.” Specifically, we state, “Under U.S. Securities and Exchange Commission rules, publicly traded companies must disclose detailed compensation packages for their ‘Named Executive Officers’ each fiscal year, which includes the Chief Executive Officer, Chief Financial Officer, and minimally the next three most highly compensated executive officers.”

Missing data and sample attrition are not concerns in the ExecuComp data set because firms are required by law to provide the data. As we explain on p. 16, “ExecuComp extracts its data directly from the annual U.S. Securities and Exchange Commission Form DEF 14A (commonly accepted as the definitive proxy statement). Under U.S. Securities and Exchange Commission rules, publicly traded companies must disclose detailed compensation packages for their ‘Named Executive Officers’ each fiscal year.”

*(2) The regressions such as using the multinomial logit model are not causal and are conditional correlation. While the authors did not claim causality, they make rather strong interpretive language ("influences," "firm leaders resist," "status uncertainty inhibits") that implies unidirectional causation without identification. The multinomial logit is a purely descriptive conditional correlation model if applied on observational data without instrumental variables, regression discontinuity, or difference-in-differences design is used. The main threat is reverse causality or simultaneity: firms that are more diversity-conscious may simultaneously be more likely to appoint minorities and more likely to already have minorities on the TMT, which could produce the observed patterns for entirely different reasons than the authors' suggested theories. If the authors are unable to provide causal evidence, the authors need to tone down their language to emphasize conditional correlation rather than using language that implies causality.*

We acknowledge that our time-lagged analyses produce conditional correlations and do not provide direct evidence of causality. We replaced language that implied causality in the title and throughout the paper when reporting empirical findings. We retained causal language in our theory and parts of the discussion that addressed the implications of the results, but we were careful to add language to emphasize uncertainty where appropriate (e.g., “our results suggest”). We also added the following to the new Limitations section to the Discussion (p. 42):

As with any research, this paper has limitations. First, it is important to acknowledge that our longitudinal analyses produce conditional correlations and do not provide direct evidence of causality. Although we address some concerns with our robustness checks that included a variety of models and controls, these analyses are vulnerable to problems related to incidental parameters, unobserved confounders, and violations of strict exogeneity. In short, the pattern of results is consistent with our theory, but our causal inferences are unsupported by the analyses.

*(3) Related to point 2, the number of firms such as Table 3 (and S2) report N in terms of firm-year observations, but the number of unique firms is never reported anywhere in the paper. For a panel data study using ExecuComp, this is quite a standard reporting expectation. Without knowing the number of firms, it is impossible to assess the degree of clustering, the effective degrees of freedom, or whether the results are driven by a small number of highly influential firms. The authors also do not cluster standard errors at the firm level in the main specification, which is a key concern.*

As mentioned above, we now report the number of unique firms in the sample in the abstract, on p. 4, and in the note under Table 3 on p. 26.

We also include a multinomial problem with selection, clustered standard errors, and year dummies. S2 Table shows that the results are essentially the same with and without the clustered standard errors. Page 22 of the revised manuscript describes all the analyses we ran to assess the robustness of the results.

*(4) The regressions do not seem to include other types of fixed effects (year, industry, state-level fixed effects, etc.) other than the firm fixed effects. While the authors do add control variables in response to reviewer comments, this may not be sufficient to capture idiosyncratic shocks that happen over time. I will suggest the authors consider including year fixed effects in their regressions at the minimum.*

S2 Table also shows that the results are essentially the same with and without year fixed effects. Again, please see p. 22 of the revised manuscript for a full list of the analyses we ran to assess the robustness of the results.

*(5) It is not clear how understanding the results is useful to business firms, industry leaders, or business policies, and where the novelty lies. The paper's contributions to organizational behavior and sociology scholarship are reasonably articulated. However, the practical managerial or business policy implications are thin. The paper documents a descriptive pattern TMT racioethnic composition consistent with maintaining the status quo, but offers little guidance for how firms, boards, or regulators could actually use the results to improve their board members’ racial heterogeneity. Moreover, since the sample of firms are profit-seeking corporate firms, what does having an ethnic-diverse TMT contribute to the firms’ returns and earnings? If firms are profitable and shareholders are happy, why should they be concerned about the racioethnicity of their TMT? The authors should provide explicit reasons and justifications why corporations should care about this.*

To address these concerns, we added a Practical Implications section (see p. 40) that explains that firm leaders face scrutiny about leadership diversity from stakeholder groups other than shareholders, even though the US federal government recently reversed its policy. It also explains when and why firm leaders should use caution when making TMT appointment decisions because the results may reflect a tendency for firm leaders to overlook the most qualified individual due to their racioethnic background rather than appoint them based on their abilities. Furthermore, we note how the current results help to draw awareness to the potential for bias, which allows firm leaders to leverage strategies that can reduce bias.

Regarding whether TMT racioethnic diversity contributes to the firms’ returns and earnings, we believe that is an important direction for future research. However, our paper focuses on the antecedents of TMT racioethnic diversity, and we believe that addressing both the antecedents and consequences of TMT racioethnic diversity is not possible in a single paper. Examining the consequences would require a completely different theory and a host of additional analyses.

*Review Comments to the Author*

*This paper has improved substantially from the previous version, and I appreciate the authors’ efforts to clarify the research question, strengthen the introduction, and provide additional discussion regarding subgroup differences. In particular, the clarification regarding the role of Boards of Directors in TMT appointments helps improve the theoretical framing of the paper. The paper continues to have significant potential. At the same time, I still have some concerns regarding the connection between the proposed theoretical mechanism and the empirical model.*

*In the revised manuscript, the authors now explain that Boards of Directors are the primary actors making TMT appointment decisions and that boards are overwhelmingly white. This clarification is helpful. However, the theoretical explanation now seems to rely much more on board-level dynamics and board-level status concerns than in the previous version, while the empirical analyses continue to focus mainly on TMT composition. As a result, there still seems to be some disconnect between the proposed mechanism and what is actually being tested empirically. Relatedly, the manuscript now argues that Boards of Directors are invested in preserving the racioethnic status quo, but board composition and board-level dynamics are not directly examined in the analyses. Thus, it remains somewhat unclear whether the observed patterns are best interpreted as evidence of board-level status uncertainty, rather than alternative explanations such as homophily, network-based recruitment, sponsorship dynamics, or broader organizational inertia. I am not suggesting that the authors necessarily need to directly test board-level mechanisms empirically. However, I do think the paper would benefit from a more explicit discussion of the extent to which the proposed mechanism is inferred rather than directly observed, as well as the limitations of the current design in distinguishing status uncertainty from alternative explanations.*

To address this issue, we now include the following paragraph in the Limitations section of the Discussion (p. 43):

Moreover, our theory is about firm leaders’ appointment decisions, but our analyses do not directly examine key aspects of our theory, including board composition and board-level dynamics. Thus, the proposed mechanism is inferred rather than directly observed. We therefore cannot completely rule out alternative explanations such as homophily, network-based recruitment, sponsorship dynamics, or broader organizational inertia.

*I also appreciate the additional discussion regarding the weaker findings for East Asians and Indians. At the same time, the current interpretation feels somewhat speculative given that these explanations are not directly tested in the paper. It may be better to frame these interpretations somewhat more cautiously as possible directions for future research.*

In the prior version of the paper, we explained that “The current data cannot address the reason for this variability, but it could be associated with how Asian Americans achieve higher levels of education, earn higher incomes, and report less (but still considerable) discrimination than other racioethnic minority groups in the United States [88-89].” To further emphasize the speculative nature of our interpretation in the revision, we added the following to p. 33: “As indicated above, this interpretation is speculative, but examining this result could be a worthwhile direction for future research.”

*Overall, I believe the manuscript has become stronger through revision, and I hope these comments are helpful in further strengthening the theoretical coherence of the paper.*
