# Supplementary material for: Existing racial and ethnic representation on top management teams predicts subsequent top management team appointments that preserve the demographic status quo
Source: PLoS One. 2026 Sep 8;21(9):e0357710. doi: 10.1371/journal.pone.0357710 (PMC13552966; doi:10.1371/journal.pone.0357710)
Supplement: Peer review history

# Peer review: Decision Letter 0

**DOI:** 10.1371/journal.pone.0357710.r001

**Authors:** Woojin Yoon

25 Sep 2025

Dear Dr. Bauman,

Thank you for submitting your manuscript to PLOS ONE. After careful consideration, we feel that it has merit but does not fully meet PLOS ONE’s publication criteria as it currently stands. Therefore, we invite you to submit a revised version of the manuscript that addresses the points raised during the review process.

Please submit your revised manuscript by Nov 09 2025 11:59PM. If you will need more time than this to complete your revisions, please reply to this message or contact the journal office at plosone@plos.org. When you're ready to submit your revision, log on to https://www.editorialmanager.com/pone/ and select the 'Submissions Needing Revision' folder to locate your manuscript file.

- A rebuttal letter that responds to each point raised by the academic editor and reviewer(s). You should upload this letter as a separate file labeled 'Response to Reviewers'.
- A marked-up copy of your manuscript that highlights changes made to the original version. You should upload this as a separate file labeled 'Revised Manuscript with Track Changes'.
- An unmarked version of your revised paper without tracked changes. You should upload this as a separate file labeled 'Manuscript'.

If you would like to make changes to your financial disclosure, please include your updated statement in your cover letter. Guidelines for resubmitting your figure files are available below the reviewer comments at the end of this letter.

If applicable, we recommend that you deposit your laboratory protocols in protocols.io to enhance the reproducibility of your results. Protocols.io assigns your protocol its own identifier (DOI) so that it can be cited independently in the future. For instructions see: https://journals.plos.org/plosone/s/submission-guidelines#loc-laboratory-protocols. Additionally, PLOS ONE offers an option for publishing peer-reviewed Lab Protocol articles, which describe protocols hosted on protocols.io. Read more information on sharing protocols at https://plos.org/protocols?utm_medium=editorial-email&utm_source=authorletters&utm_campaign=protocols.

We look forward to receiving your revised manuscript.

Kind regards,

Woojin Yoon

Academic Editor

PLOS ONE

**Journal Requirements:**

1. When submitting your revision, we need you to address these additional requirements.

Please ensure that your manuscript meets PLOS ONE's style requirements, including those for file naming. The PLOS ONE style templates can be found at

https://journals.plos.org/plosone/s/file?id=wjVg/PLOSOne_formatting_sample_main_body.pdf and

https://journals.plos.org/plosone/s/file?id=ba62/PLOSOne_formatting_sample_title_authors_affiliations.pdf

2. Thank you for uploading your study's underlying data set. Unfortunately, the repository you have noted in your Data Availability statement does not qualify as an acceptable data repository according to PLOS's standards.

At this time, please upload the minimal data set necessary to replicate your study's findings to a stable, public repository (such as figshare or Dryad) and provide us with the relevant URLs, DOIs, or accession numbers that may be used to access these data. For a list of recommended repositories and additional information on PLOS standards for data deposition, please see https://journals.plos.org/plosone/s/recommended-repositories.

3. In the online submission form, you indicated that your data will be submitted to a repository upon acceptance.  We strongly recommend all authors deposit their data before acceptance, as the process can be lengthy and hold up publication timelines. Please note that, though access restrictions are acceptable now, your entire minimal  dataset will need to be made freely accessible if your manuscript is accepted for publication. This policy applies to all data except where public deposition would breach compliance with the protocol approved by your research ethics board. If you are unable to adhere to our open data policy, please kindly revise your statement to explain your reasoning and we will seek the editor's input on an exemption.

4. When completing the data availability statement of the submission form, you indicated that you will make your data available on acceptance. We strongly recommend all authors decide on a data sharing plan before acceptance, as the process can be lengthy and hold up publication timelines. Please note that, though access restrictions are acceptable now, your entire data will need to be made freely accessible if your manuscript is accepted for publication. This policy applies to all data except where public deposition would breach compliance with the protocol approved by your research ethics board. If you are unable to adhere to our open data policy, please kindly revise your statement to explain your reasoning and we will seek the editor's input on an exemption. Please be assured that, once you have provided your new statement, the assessment of your exemption will not hold up the peer review process.

5. Please include your full ethics statement in the ‘Methods’ section of your manuscript file. In your statement, please include the full name of the IRB or ethics committee who approved or waived your study, as well as whether or not you obtained informed written or verbal consent. If consent was waived for your study, please include this information in your statement as well.

6. We notice that your supplementary tables are included in the manuscript file. Please remove them and upload them with the file type 'Supporting Information'. Please ensure that each Supporting Information file has a legend listed in the manuscript after the references list.

[Note: HTML markup is below. Please do not edit.]

Reviewers' comments:

Reviewer's Responses to Questions

**Comments to the Author**

1. Is the manuscript technically sound, and do the data support the conclusions?

Reviewer #1: Partly

**********

2. Has the statistical analysis been performed appropriately and rigorously?

Reviewer #1: I Don't Know

**********

3. Have the authors made all data underlying the findings in their manuscript fully available?

The PLOS Data policy

Reviewer #1: Yes

**********

4. Is the manuscript presented in an intelligible fashion and written in standard English?

Reviewer #1: Yes

**********

**Reviewer #1:** This paper investigated whether existing racial/ethnic composition of TMT influences later appointment of TMT members. The authors shed light on status concerns to induce the tendency of appointment decisions. This paper has significant potential. At the same time, I have some concerns about both theory and methods; I hope my comments will be useful to authors to push the manuscript forward.

1. From a theoretical standpoint, it is not well explained why CEO and other TMT members show different patterns. Like CEO, other TMT members, especially those in minority groups, may want to bring in more minorities whether it is because they feel emotionally connected or because they need more political capital. Thus, by extension, the authors need to explain why individuals care more about the group-level status quo than their personal preferences. Along similar lines, it seems less convincing that the current explanation assumes that TMT members want to preserve status quo and that it is the best thing to do. Although the authors describe this as status quo bias, but it needs to be explained further.

2. The front part of the current manuscript does not appear to guide the audiences to understand the research question and why it is important. I suggest for the authors to develop a clear research question which the authors can use to guide the research.

3. The efforts and methods to identify the racioethnicity of the TMT members are vey impressive, but there is very limited information about the firms to which the TMT belongs, other than ROA and Q. For example, firm-level covariates such as ownership structure, strategic priorities, corporate culture can have a very significant impact on TMT appointments and should be controlled for. Similarly, industry-level information such as industry dynamics and market conditions need to be controlled for.

4. As the authors note, the detailed classification of racioethnicity is a huge strength of the paper. However, the interpretation is lacking in the explanation of each racioethnicity individually. For example, there needs to be more explanation as to why East Asians and Indians do not show similar patterns to other minority groups.

**********

what does this mean?). If published, this will include your full peer review and any attached files.

If you choose “no”, your identity will remain anonymous but your review may still be made public.

**Do you want your identity to be public for this peer review?**  For information about this choice, including consent withdrawal, please see our Privacy Policy

Reviewer #1: No

**********

[NOTE: If reviewer comments were submitted as an attachment file, they will be attached to this email and accessible via the submission site. Please log into your account, locate the manuscript record, and check for the action link "View Attachments". If this link does not appear, there are no attachment files.]

While revising your submission, please upload your figure files to the Preflight Analysis and Conversion Engine (PACE) digital diagnostic tool, https://pacev2.apexcovantage.com/. PACE helps ensure that figures meet PLOS requirements. To use PACE, you must first register as a user. Registration is free. Then, login and navigate to the UPLOAD tab, where you will find detailed instructions on how to use the tool. If you encounter any issues or have any questions when using PACE, please email PLOS at figures@plos.org. Please note that Supporting Information files do not need this step.

# Author comment: Author response to Decision Letter 1

**DOI:** 10.1371/journal.pone.0357710.r002

30 Apr 2026

Dear Woojin Yoon:

Thank you very much for the opportunity to revise our manuscript. I apologize for the additional time it took us to submit the revision, but two of us have been dealing with some major health issues. However, we now have carefully engaged with all the feedback, and we believe that the paper is much stronger as a result. We hope you agree.

We address each point raised by you and the reviewer below:

Journal Requirements:

1. Please ensure that your manuscript meets PLOS ONE's style requirements, including those for file naming.

We carefully formatted all materials. Please accept our apologies for failing to conform to the journal’s standards in our initial submission.

2. Thank you for uploading your study's underlying data set. Unfortunately, the repository you have noted in your Data Availability statement does not qualify as an acceptable data repository according to PLOS's standards.

We uploaded our data to OSF: https://osf.io/2wgrf/overview?view_only=dc1d4102ddaf41a090cd6d87211df996

3. In the online submission form, you indicated that your data will be submitted to a repository upon acceptance. We strongly recommend all authors deposit their data before acceptance, as the process can be lengthy and hold up publication timelines. Please note that, though access restrictions are acceptable now, your entire minimal dataset will need to be made freely accessible if your manuscript is accepted for publication. This policy applies to all data except where public deposition would breach compliance with the protocol approved by your research ethics board. If you are unable to adhere to our open data policy, please kindly revise your statement to explain your reasoning and we will seek the editor's input on an exemption.

We uploaded our data to OSF:

https://osf.io/2wgrf/overview?view_only=dc1d4102ddaf41a090cd6d87211df996

4. When completing the data availability statement of the submission form, you indicated that you will make your data available on acceptance. We strongly recommend all authors decide on a data sharing plan before acceptance, as the process can be lengthy and hold up publication timelines. Please note that, though access restrictions are acceptable now, your entire data will need to be made freely accessible if your manuscript is accepted for publication. This policy applies to all data except where public deposition would breach compliance with the protocol approved by your research ethics board. If you are unable to adhere to our open data policy, please kindly revise your statement to explain your reasoning and we will seek the editor's input on an exemption. Please be assured that, once you have provided your new statement, the assessment of your exemption will not hold up the peer review process.

We uploaded our data to OSF:

https://osf.io/2wgrf/overview?view_only=dc1d4102ddaf41a090cd6d87211df996

5. Please include your full ethics statement in the ‘Methods’ section of your manuscript file. In your statement, please include the full name of the IRB or ethics committee who approved or waived your study, as well as whether or not you obtained informed written or verbal consent. If consent was waived for your study, please include this information in your statement as well.

On p. 15 of the manuscript, we now make the following declaration:

The Institutional Review Board at the University of California, Irvine does not require and will not provide approval for this research because it involves secondary uses of identifiable private information that is publicly available and external of the University (Exempt Category 4). For the same reason, informed consent was not required.

6. We notice that your supplementary tables are included in the manuscript file. Please remove them and upload them with the file type 'Supporting Information'. Please ensure that each Supporting Information file has a legend listed in the manuscript after the references list.

We submitted the supplementary tables in the appropriate format and include a legend list in the manuscript after the references list.

Reviewer #1: This paper investigated whether existing racial/ethnic composition of TMT influences later appointment of TMT members. The authors shed light on status concerns to induce the tendency of appointment decisions. This paper has significant potential. At the same time, I have some concerns about both theory and methods; I hope my comments will be useful to authors to push the manuscript forward.

1. From a theoretical standpoint, it is not well explained why CEO and other TMT members show different patterns. Like CEO, other TMT members, especially those in minority groups, may want to bring in more minorities whether it is because they feel emotionally connected or because they need more political capital. Thus, by extension, the authors need to explain why individuals care more about the group-level status quo than their personal preferences. Along similar lines, it seems less convincing that the current explanation assumes that TMT members want to preserve status quo and that it is the best thing to do. Although the authors describe this as status quo bias, but it needs to be explained further.

In the prior draft of the paper, we used the term “firm leaders” when referring to the agents who make TMT appointments, and our failure to define this term left open opportunities for misunderstanding. We now explicitly state on p. 3 that Boards of Directors have the formal power to make TMT appointments, although other parties may influence the decision (e.g., CEOs, large investors). We hope this description of the corporate governance structure eliminates confusion about who makes TMT appointments. This governance structure is important because Boards of Directors of companies in our sample were overwhelmingly white; the 2016 Board Diversity Census found that 87.2% of board members of Fortune 500 companies were white (Alliance for Board Diversity, Deloitte). Thus, it is the overwhelmingly white Board of Directors that is invested in the group-level status quo (not the CEO or other TMT members).

Our results indicate that firm leaders—especially the Board of Directors—is invested in the racioethnic status quo because they resist adding additional members of a racioethnic minority group that is already represented on the TMT and they also are much more likely to appoint a racioethnic minority group member when a member of the same group recently departed the TMT.

Our results also suggest that CEO racioethnicity has a different influence on appointment decisions than the overall racioethnic composition of the TMT, and we provide on p. 14 three reasons why this may be the case. In short, we argue that the effect of CEO racioethnicity differs because (1) CEOs who are racioethnic minority group members are in a strong position to advocate for members of their own racioethnic minority group to join the TMT, (2) CEOs who are racioethnic minority group members should be less invested in limiting opportunities for members of their own racioethnicity group, and (3) candidates from the same racioethnic minority group as the CEO may be more attracted to the role.

2. The front part of the current manuscript does not appear to guide the audiences to understand the research question and why it is important. I suggest for the authors to develop a clear research question which the authors can use to guide the research.

We made several revisions to the introduction to be more explicit about our research question and how it guided our research. For example, we now begin the second paragraph with the following: “We examine how the existing racioethnic composition of TMTs influences subsequent appointments of racioethnic minority group members to TMTs to shed light on why TMTs remain relatively homogeneous in terms of racioethnicity despite widespread institutional pressure to increase diversity (e.g., Hunt et al. 2015; Richard, Triana, & Li 2021; Ely & Thomas 2020).” We then contrast our approach and our theory with prior research on the topic, briefly review our results, and finish by identifying four main contributions to the literature.

3. The efforts and methods to identify the racioethnicity of the TMT members are vey impressive, but there is very limited information about the firms to which the TMT belongs, other than ROA and Q. For example, firm-level covariates such as ownership structure, strategic priorities, corporate culture can have a very significant impact on TMT appointments and should be controlled for. Similarly, industry-level information such as industry dynamics and market conditions need to be controlled for.

We appreciate the suggestion given that the ExecuComp database is large and includes all S&P 1500 firms plus companies that were once part of the 1500 index and are still trading. We added several firm-level covariates to our analyses, including TMT size, firm size (# of employees), leverage, CAPX/sales, diversity of the TMT (as an index of corporate culture), institutional ownership, and industry dynamism. Few of these factors had any significant effect and none had systematic effects for any racioethnic group. In short, our results remain effectively the same with the additional controls included in the analyses.

4. As the authors note, the detailed classification of racioethnicity is a huge strength of the paper. However, the interpretation is lacking in the explanation of each racioethnicity individually. For example, there needs to be more explanation as to why East Asians and Indians do not show similar patterns to other minority groups.

Unfortunately, our data cannot speak to why representation of East Asians and Indians had less negative effects on subsequent TMT appointments than representation of other racioethnic minority groups, but we agree that it was a mistake not to address these results directly in the discussion. We added the following the pp. 34-35:

Before moving on, it is important to note that although the results were remarkably consistent across racioethnic groups and analyses, there was some variability. In particular, the effects of having East Asians and Indians on the TMT did not significantly reduce future TMT appointments of East Asians and Indians, respectively, even though the parameter estimates for all racioethnic minority groups, including East Asians and Indians were negative. The current data cannot address the reason for this variability, but it could be associated with how Asian Americans achieve higher levels of education, earn higher incomes, and report less (but still considerable) discrimination than other racioethnic minority groups in the United States (Hirshman & Wong, 1986; McMurtry et al. 2019). In any case, these results underscore how each social group has a unique history and set of contemporary challenges that may influence the way firm leaders and the general public conceptualize diversity and respond to issues involving a particular group (Sidanius & Pratto, 1999). Even though researchers commonly aggregate across racioethnic minority groups, numerical minority status in a given context is just one of potentially many characteristics of any group (and not necessarily the most distinctive one). Therefore, future research should continue to test for and seek to understand differences across racioethnic minority groups.

Attachment Submitted filename: Response to Reviewers PLOS.docx

# Peer review: Decision Letter 1

**DOI:** 10.1371/journal.pone.0357710.r003

**Authors:** Soo Keong Yong

19 May 2026

Dear Dr. Bauman,

Thank you for submitting your manuscript to PLOS ONE. After careful consideration, we feel that it has merit but does not fully meet PLOS ONE’s publication criteria as it currently stands. Therefore, we invite you to submit a revised version of the manuscript that addresses the points raised during the review process.

In addition, please kindly note the*further comments from the previous reviewer*  displayed below.

Please submit your revised manuscript by Jul 03 2026 11:59PM. If you will need more time than this to complete your revisions, please reply to this message or contact the journal office at plosone@plos.org. When you're ready to submit your revision, log on to https://www.editorialmanager.com/pone/ and select the 'Submissions Needing Revision' folder to locate your manuscript file.

Please include the following items when submitting your revised manuscript:

- A letter that responds to each point raised by the academic editor and reviewer(s). You should upload this letter as a separate file labeled 'Response to Reviewers'.
- A marked-up copy of your manuscript that highlights changes made to the original version. You should upload this as a separate file labeled 'Revised Manuscript with Track Changes'.
- An unmarked version of your revised paper without tracked changes. You should upload this as a separate file labeled 'Manuscript'.

If you would like to make changes to your financial disclosure, please include your updated statement in your cover letter. Guidelines for resubmitting your figure files are available below the reviewer comments at the end of this letter.

If applicable, we recommend that you deposit your laboratory protocols in protocols.io to enhance the reproducibility of your results. Protocols.io assigns your protocol its own identifier (DOI) so that it can be cited independently in the future. For instructions see: https://journals.plos.org/plosone/s/submission-guidelines#loc-laboratory-protocols. Additionally, PLOS ONE offers an option for publishing peer-reviewed Lab Protocol articles, which describe protocols hosted on protocols.io. Read more information on sharing protocols at https://plos.org/protocols?utm_medium=editorial-email&utm_source=authorletters&utm_campaign=protocols.

As the corresponding author, your ORCID iD is verified in the submission system and will appear in the published article. PLOS supports the use of ORCID, and we encourage all coauthors to register for an ORCID iD and use it as well. Please encourage your coauthors to verify their ORCID iD within the submission system before final acceptance, as unverified ORCID iDs will not appear in the published article. *Only*  the individual author can complete the verification step; PLOS staff *cannot*  verify ORCID iDs on behalf of authors.

We look forward to receiving your revised manuscript.

Kind regards,

Soo Keong Yong, Ph.D.

Academic Editor

PLOS One

Journal Requirements:

If the reviewer comments include a recommendation to cite specific previously published works, please review and evaluate these publications to determine whether they are relevant and should be cited. There is no requirement to cite these works unless the editor has indicated otherwise.

Additional Editor Comments:

(1) The data description is not sufficiently transparent, specifically how the data are being cleaned and used in the regression analysis, is not clear. For example, the paper did not describe the total number of unique firms in the sample (it only reports person-firm-years), and it does not describe how ExecuComp defines "top managers". In addition, there is no description of how missing data and sample attrition – which is common in micro firm-level data – is being managed and documented.

(2) The regressions such as using the multinomial logit model are not causal and are conditional correlation. While the authors did not claim causality, they make rather strong interpretive language ("influences," "firm leaders resist," "status uncertainty inhibits") that implies unidirectional causation without identification. The multinomial logit is a purely descriptive conditional correlation model if applied on observational data without instrumental variables, regression discontinuity, or difference-in-differences design is used. The main threat is reverse causality or simultaneity: firms that are more diversity-conscious may simultaneously be more likely to appoint minorities and more likely to already have minorities on the TMT, which could produce the observed patterns for entirely different reasons than the authors' suggested theories. If the authors are unable to provide causal evidence, the authors need to tone down their language to emphasize conditional correlation rather than using language that implies causality.

(3) Related to point 2, the number of firms such as Table 3 (and S2) report N in terms of firm-year observations, but the number of unique firms is never reported anywhere in the paper. For a panel data study using ExecuComp, this is quite a standard reporting expectation. Without knowing the number of firms, it is impossible to assess the degree of clustering, the effective degrees of freedom, or whether the results are driven by a small number of highly influential firms. The authors also do not cluster standard errors at the firm level in the main specification, which is a key concern.

(4) The regressions do not seem to include other types of fixed effects (year, industry, state-level fixed effects, etc.) other than the firm fixed effects. While the authors do add control variables in response to reviewer comments, this may not be sufficient to capture idiosyncratic shocks that happen over time. I will suggest the authors consider including year fixed effects in their regressions at the minimum.

(5) It is not clear how understanding the results is useful to business firms, industry leaders, or business policies, and where the novelty lies. The paper's contributions to organizational behavior and sociology scholarship are reasonably articulated. However, the practical managerial or business policy implications are thin. The paper documents a descriptive pattern TMT racioethnic composition consistent with maintaining the status quo, but offers little guidance for how firms, boards, or regulators could actually use the results to improve their board members’ racial heterogeneity. Moreover, since the sample of firms are profit-seeking corporate firms, what does having an ethnic-diverse TMT contribute to the firms’ returns and earnings? If firms are profitable and shareholders are happy, why should they be concerned about the racioethnicity of their TMT? The authors should provide explicit reasons and justifications why corporations should care about this.

[Note: HTML markup is below. Please do not edit.]

Reviewers' comments:

Reviewer's Responses to Questions

**Comments to the Author**

Reviewer #1: (No Response)

**********

2. Is the manuscript technically sound, and do the data support the conclusions?

Reviewer #1: Partly

**********

3. Has the statistical analysis been performed appropriately and rigorously?

Reviewer #1: Yes

**********

4. Have the authors made all data underlying the findings in their manuscript fully available?

The PLOS Data policy

Reviewer #1: No

**********

5. Is the manuscript presented in an intelligible fashion and written in standard English?

Reviewer #1: Yes

**********

Reviewer #1: This paper has improved substantially from the previous version, and I appreciate the authors’ efforts to clarify the research question, strengthen the introduction, and provide additional discussion regarding subgroup differences. In particular, the clarification regarding the role of Boards of Directors in TMT appointments helps improve the theoretical framing of the paper. The paper continues to have significant potential.

At the same time, I still have some concerns regarding the connection between the proposed theoretical mechanism and the empirical model.

In the revised manuscript, the authors now explain that Boards of Directors are the primary actors making TMT appointment decisions and that boards are overwhelmingly white. This clarification is helpful. However, the theoretical explanation now seems to rely much more on board-level dynamics and board-level status concerns than in the previous version, while the empirical analyses continue to focus mainly on TMT composition. As a result, there still seems to be some disconnect between the proposed mechanism and what is actually being tested empirically.

Relatedly, the manuscript now argues that Boards of Directors are invested in preserving the racioethnic status quo, but board composition and board-level dynamics are not directly examined in the analyses. Thus, it remains somewhat unclear whether the observed patterns are best interpreted as evidence of board-level status uncertainty, rather than alternative explanations such as homophily, network-based recruitment, sponsorship dynamics, or broader organizational inertia.

I am not suggesting that the authors necessarily need to directly test board-level mechanisms empirically. However, I do think the paper would benefit from a more explicit discussion of the extent to which the proposed mechanism is inferred rather than directly observed, as well as the limitations of the current design in distinguishing status uncertainty from alternative explanations.

I also appreciate the additional discussion regarding the weaker findings for East Asians and Indians. At the same time, the current interpretation feels somewhat speculative given that these explanations are not directly tested in the paper. It may be better to frame these interpretations somewhat more cautiously as possible directions for future research.

Overall, I believe the manuscript has become stronger through revision, and I hope these comments are helpful in further strengthening the theoretical coherence of the paper.

**********

what does this mean?). If published, this will include your full peer review and any attached files.

If you choose “no”, your identity will remain anonymous but your review may still be made public.

**Do you want your identity to be public for this peer review?**  For information about this choice, including consent withdrawal, please see our Privacy Policy

Reviewer #1: No

**********

[NOTE: If reviewer comments were submitted as an attachment file, they will be attached to this email and accessible via the submission site. Please log into your account, locate the manuscript record, and check for the action link "View Attachments". If this link does not appear, there are no attachment files.]

To ensure your figures meet our technical requirements, please review our figure guidelines: https://journals.plos.org/plosone/s/figures

You may also use PLOS’s free figure tool, NAAS, to help you prepare publication quality figures: https://journals.plos.org/plosone/s/figures#loc-tools-for-figure-preparation.

NAAS will assess whether your figures meet our technical requirements by comparing each figure against our figure specifications.

# Author comment: Author response to Decision Letter 2

**DOI:** 10.1371/journal.pone.0357710.r004

20 Jul 2026

PONE-D-24-54283

Existing racial and ethnic representation on top management teams predicts subsequent top management team appointments that preserve the demographic status quo

Dear Soo Keong Yong:

Thank you very much for the opportunity to revise our manuscript. We carefully engaged with the reviewer’s comments, and we believe that the paper is clearer as a result. We hope you agree.

Editor Comments:

(1) The data description is not sufficiently transparent, specifically how the data are being cleaned and used in the regression analysis, is not clear. For example, the paper did not describe the total number of unique firms in the sample (it only reports person-firm-years), and it does not describe how ExecuComp defines "top managers". In addition, there is no description of how missing data and sample attrition – which is common in micro firm-level data – is being managed and documented.

Thank you for identifying ways we could improve transparency in the our description of our data.

We now report the number of unique firms in the sample in the abstract, main text on p. 4, and note under Table 3 on p. 26.

We now explain on p. 16 in the data construction section of the manuscript how ExecuComp defines “top managers.” Specifically, we state, “Under U.S. Securities and Exchange Commission rules, publicly traded companies must disclose detailed compensation packages for their ‘Named Executive Officers’ each fiscal year, which includes the Chief Executive Officer, Chief Financial Officer, and minimally the next three most highly compensated executive officers.”

Missing data and sample attrition are not concerns in the ExecuComp data set because firms are required by law to provide the data. As we explain on p. 16, “ExecuComp extracts its data directly from the annual U.S. Securities and Exchange Commission Form DEF 14A (commonly accepted as the definitive proxy statement). Under U.S. Securities and Exchange Commission rules, publicly traded companies must disclose detailed compensation packages for their ‘Named Executive Officers’ each fiscal year.”

(2) The regressions such as using the multinomial logit model are not causal and are conditional correlation. While the authors did not claim causality, they make rather strong interpretive language ("influences," "firm leaders resist," "status uncertainty inhibits") that implies unidirectional causation without identification. The multinomial logit is a purely descriptive conditional correlation model if applied on observational data without instrumental variables, regression discontinuity, or difference-in-differences design is used. The main threat is reverse causality or simultaneity: firms that are more diversity-conscious may simultaneously be more likely to appoint minorities and more likely to already have minorities on the TMT, which could produce the observed patterns for entirely different reasons than the authors' suggested theories. If the authors are unable to provide causal evidence, the authors need to tone down their language to emphasize conditional correlation rather than using language that implies causality.

We acknowledge that our time-lagged analyses produce conditional correlations and do not provide direct evidence of causality. We replaced language that implied causality in the title and throughout the paper when reporting empirical findings. We retained causal language in our theory and parts of the discussion that addressed the implications of the results, but we were careful to add language to emphasize uncertainty where appropriate (e.g., “our results suggest”). We also added the following to the new Limitations section to the Discussion (p. 42):

As with any research, this paper has limitations. First, it is important to acknowledge that our longitudinal analyses produce conditional correlations and do not provide direct evidence of causality. Although we address some concerns with our robustness checks that included a variety of models and controls, these analyses are vulnerable to problems related to incidental parameters, unobserved confounders, and violations of strict exogeneity. In short, the pattern of results is consistent with our theory, but our causal inferences are unsupported by the analyses.

(3) Related to point 2, the number of firms such as Table 3 (and S2) report N in terms of firm-year observations, but the number of unique firms is never reported anywhere in the paper. For a panel data study using ExecuComp, this is quite a standard reporting expectation. Without knowing the number of firms, it is impossible to assess the degree of clustering, the effective degrees of freedom, or whether the results are driven by a small number of highly influential firms. The authors also do not cluster standard errors at the firm level in the main specification, which is a key concern.

As mentioned above, we now report the number of unique firms in the sample in the abstract, on p. 4, and in the note under Table 3 on p. 26.

We also include a multinomial problem with selection, clustered standard errors, and year dummies. S2 Table shows that the results are essentially the same with and without the clustered standard errors. Page 22 of the revised manuscript describes all the analyses we ran to assess the robustness of the results.

(4) The regressions do not seem to include other types of fixed effects (year, industry, state-level fixed effects, etc.) other than the firm fixed effects. While the authors do add control variables in response to reviewer comments, this may not be sufficient to capture idiosyncratic shocks that happen over time. I will suggest the authors consider including year fixed effects in their regressions at the minimum.

S2 Table also shows that the results are essentially the same with and without year fixed effects. Again, please see p. 22 of the revised manuscript for a full list of the analyses we ran to assess the robustness of the results.

(5) It is not clear how understanding the results is useful to business firms, industry leaders, or business policies, and where the novelty lies. The paper's contributions to organizational behavior and sociology scholarship are reasonably articulated. However, the practical managerial or business policy implications are thin. The paper documents a descriptive pattern TMT racioethnic composition consistent with maintaining the status quo, but offers little guidance for how firms, boards, or regulators could actually use the results to improve their board members’ racial heterogeneity. Moreover, since the sample of firms are profit-seeking corporate firms, what does having an ethnic-diverse TMT contribute to the firms’ returns and earnings? If firms are profitable and shareholders are happy, why should they be concerned about the racioethnicity of their TMT? The authors should provide explicit reasons and justifications why corporations should care about this.

To address these concerns, we added a Practical Implications section (see p. 40) that explains that firm leaders face scrutiny about leadership diversity from stakeholder groups other than shareholders, even though the US federal government recently reversed its policy. It also explains when and why firm leaders should use caution when making TMT appointment decisions because the results may reflect a tendency for firm leaders to overlook the most qualified individual due to their racioethnic background rather than appoint them based on their abilities. Furthermore, we note how the current results help to draw awareness to the potential for bias, which allows firm leaders to leverage strategies that can reduce bias.

Regarding whether TMT racioethnic diversity contributes to the firms’ returns and earnings, we believe that is an important direction for future research. However, our paper focuses on the antecedents of TMT racioethnic diversity, and we believe that addressing both the antecedents and consequences of TMT racioethnic diversity is not possible in a single paper. Examining the consequences would require a completely different theory and a host of additional analyses.

Review Comments to the Author

This paper has improved substantially from the previous version, and I appreciate the authors’ efforts to clarify the research question, strengthen the introduction, and provide additional discussion regarding subgroup differences. In particular, the clarification regarding the role of Boards of Directors in TMT appointments helps improve the theoretical framing of the paper. The paper continues to have significant potential. At the same time, I still have some concerns regarding the connection between the proposed theoretical mechanism and the empirical model.

In the revised manuscript, the authors now explain that Boards of Directors are the primary actors making TMT appointment decisions and that boards are overwhelmingly white. This clarification is helpful. However, the theoretical explanation now seems to rely much more on board-level dynamics and board-level status concerns than in the previous version, while the empirical analyses continue to focus mainly on TMT composition. As a result, there still seems to be some disconnect between the proposed mechanism and what is actually being tested empirically. Relatedly, the manuscript now argues that Boards of Directors are invested in preserving the racioethnic status quo, but board composition and board-level dynamics are not directly examined in the analyses. Thus, it remains somewhat unclear whether the observed patterns are best interpreted as evidence of board-level status uncertainty, rather than alternative explanations such as homophily, network-based recruitment, sponsorship dynamics, or broader organizational inertia. I am not suggesting that the authors necessarily need to directly test board-level mechanisms empirically. However, I do think the paper would benefit from a more explicit discussion of the extent to which the proposed mechanism is inferred rather than directly observed, as well as the limitations of the current design in distinguishing status uncertainty from alternative explanations.

To address this issue, we now include the following paragraph in the Limitations section of the Discussion (p. 43):

Moreover, our theory is about firm leaders’ appointment decisions, but our analyses do not directly examine key aspects of our theory, including board composition and board-level dynamics. Thus, the proposed mechanism is inferred rather than directly observed. We therefore cannot completely rule out alternative explanations such as homophily, network-based recruitment, sponsorship dynamics, or broader organizational inertia.

I also appreciate the additional discussion regarding the weaker findings for East Asians and Indians. At the same time, the current interpretation feels somewhat speculative given that these explanations are not directly tested in the paper. It may be better to frame these interpretations somewhat more cautiously as possible directions for future research.

In the prior version of the paper, we explained that “The current data cannot address the reason for this variability, but it could be associated with how Asian Americans achieve higher levels of education, earn higher incomes, and report less (but still considerable) discrimination than other racioethnic minority groups in the United States [88-89].” To further emphasize the speculative nature of our interpretation in the revision, we added the following to p. 33: “As indicated above, this interpretation is speculative, but examining this result could be a worthwhile direction for future research.”

Overall, I believe the manuscript has become stronger through revision, and I hope these comments are helpful in further strengthening the theoretical coherence of the paper.

Attachment Submitted filename: Response to Reviewers PLOS (R2).docx

# Peer review: Decision Letter 2

**DOI:** 10.1371/journal.pone.0357710.r005

**Authors:** Soo Keong Yong

21 Aug 2026

Existing racial and ethnic representation on top management teams predicts subsequent top management team appointments that preserve the demographic status quo

PONE-D-24-54283R2

Dear Dr. Bauman,

We’re pleased to inform you that your manuscript has been judged scientifically suitable for publication and will be formally accepted for publication once it meets all outstanding technical requirements.

Within one week, you’ll receive an e-mail detailing the required amendments. When these have been addressed, you’ll receive a formal acceptance letter and your manuscript will be scheduled for publication.

An invoice will be generated when your article is formally accepted. Please note, if your institution has a publishing partnership with PLOS and your article meets the relevant criteria, all or part of your publication costs will be covered. Please make sure your user information is up-to-date by logging into Editorial Manager at Editorial Manager® and clicking the ‘Update My Information' link at the top of the page. For questions related to billing, please contact billing support.

If your institution or institutions have a press office, please notify them about your upcoming paper to help maximize its impact. If they’ll be preparing press materials, please inform our press team as soon as possible -- no later than 48 hours after receiving the formal acceptance. Your manuscript will remain under strict press embargo until 2 pm Eastern Time on the date of publication. For more information, please contact onepress@plos.org.

Kind regards,

Soo Keong Yong, Ph.D.

Academic Editor

PLOS One

Additional Editor Comments (optional):

Reviewers' comments:

Reviewer's Responses to Questions

**Comments to the Author**

Reviewer #1: All comments have been addressed

**********

2. Is the manuscript technically sound, and do the data support the conclusions?

Reviewer #1: Yes

**********

3. Has the statistical analysis been performed appropriately and rigorously?

Reviewer #1: Yes

**********

4. Have the authors made all data underlying the findings in their manuscript fully available?

The PLOS Data policy

Reviewer #1: Yes

**********

5. Is the manuscript presented in an intelligible fashion and written in standard English?

Reviewer #1: Yes

**********

Reviewer #1: The authors have addressed the majority of my previous concerns satisfactorily. In particular, the revised manuscript is clearer about the role of boards in top management team appointments, appropriately acknowledges that the proposed board-level mechanism is inferred rather than directly observed, and more cautiously frames the interpretation of subgroup differences. These revisions have improved the theoretical coherence of the manuscript and better align the interpretation of the findings with the empirical design.

Overall, I believe the manuscript has improved substantially through the revision process. While some limitations necessarily remain, the authors now acknowledge them appropriately, and I do not view them as obstacles to publication. In my view, the major concerns raised in the previous round have been sufficiently addressed, and I believe the manuscript is now suitable for publication.

**********

what does this mean?). If published, this will include your full peer review and any attached files.

If you choose “no”, your identity will remain anonymous but your review may still be made public.

**Do you want your identity to be public for this peer review?**  For information about this choice, including consent withdrawal, please see our Privacy Policy

Reviewer #1: **Yes:** Yisook Lim

**********

# Editor report: Acceptance letter

**DOI:** 10.1371/journal.pone.0357710.r006

**Authors:** Soo Keong Yong

PONE-D-24-54283R2

PLOS One

Dear Dr. Bauman,

I'm pleased to inform you that your manuscript has been deemed suitable for publication in PLOS One. Congratulations! Your manuscript is now being handed over to our production team.

At this stage, our production department will prepare your paper for publication. This includes ensuring the following:

* All references, tables, and figures are properly cited

* All relevant supporting information is included in the manuscript submission,

* There are no issues that prevent the paper from being properly typeset

You will receive further instructions from the production team, including instructions on how to review your proof when it is ready. Please keep in mind that we are working through a large volume of accepted articles, so please give us a few days to review your paper and let you know the next and final steps.

Lastly, if your institution or institutions have a press office, please let them know about your upcoming paper now to help maximize its impact. If they'll be preparing press materials, please inform our press team within the next 48 hours. Your manuscript will remain under strict press embargo until 2 pm Eastern Time on the date of publication. For more information, please contact onepress@plos.org.

You will receive an invoice from PLOS for your publication fee after your manuscript has reached the completed accept phase. If you receive an email requesting payment before acceptance or for any other service, this may be a phishing scheme. Learn how to identify phishing emails and protect your accounts at https://explore.plos.org/phishing.

If we can help with anything else, please email us at customercare@plos.org.

Thank you for submitting your work to PLOS One and supporting open access.

Kind regards,

PLOS One Editorial Office Staff

on behalf of

Dr. Soo Keong Yong

Academic Editor

PLOS One
